# Experimental investigations of control principles of involuntary movement: a comprehensive review of the Kohnstamm phenomenon

**DOI:** 10.1007/s00221-017-4950-3

**Published:** 2017-04-03

**Authors:** Jack De Havas, Hiroaki Gomi, Patrick Haggard

**Affiliations:** 1 Institute of Cognitive Neuroscience, University College London, Alexandra House, 17 Queen Square, London, WC1N 3AR UK; 2 NTT Communication Science Laboratories, Nippon Telegraph and Telephone Corporation, Wakamiya 3-1, Morinosato, Atsugi, Kanagawa-Pref. 243-0198 Japan

**Keywords:** Involuntary movement, Action inhibition, Posture, Aftercontraction, Muscle afferents, Action awareness

## Abstract

The Kohnstamm phenomenon refers to the observation that if one pushes the arm hard outwards against a fixed surface for about 30 s, and then moves away from the surface and relaxes, an involuntary movement of the arm occurs, accompanied by a feeling of lightness. Central, peripheral and hybrid theories of the Kohnstamm phenomenon have been advanced. Afferent signals may be irrelevant if purely central theories hold. Alternatively, according to peripheral accounts, altered afferent signalling actually drives the involuntary movement. Hybrid theories suggest afferent signals control a centrally-programmed aftercontraction via negative position feedback control or positive force feedback control. The Kohnstamm phenomenon has provided an important scientific method for comparing voluntary with involuntary movement, both with respect to subjective experience, and for investigating whether involuntary movements can be brought under voluntary control. A full review of the literature reveals that a hybrid model best explains the Kohnstamm phenomenon. On this model, a central adaptation interacts with afferent signals at multiple levels of the motor hierarchy. The model assumes that a Kohnstamm generator sends output via the same pathways as voluntary movement, yet the resulting movement feels involuntary due to a lack of an efference copy to cancel against sensory inflow. This organisation suggests the Kohnstamm phenomenon could represent an amplification of neuromotor processes normally involved in automatic postural maintenance. Future work should determine which afferent signals contribute to the Kohnstamm phenomenon, the location of the Kohnstamm generator, and the principle of feedback control operating during the aftercontraction.

## Introduction

Developing an understanding of the involuntary mechanisms of motor control is a primary aim of motor control science. Historically, most research has focussed on involuntary responses to transient perturbations (Marsden et al. 1976b; Feldman et al. 1998; Archambault et al. 2005), and most experimental models involve brief involuntary reflex responses (Matthews 1991). These approaches encourage the view of involuntary movement as a single, discrete feedforward event, rather than an ongoing form of continuous control, occurring below the level of conscious volition. In particular, the ongoing principle of control of the involuntary movement cannot easily be assessed from brief responses. The Kohnstamm phenomenon offers a unique means to study involuntary movement free from the constraints imposed by short, transient reflex responses. We show how studying involuntary movements at this longer timescale can reveal fundamental control principles underlying human movements, both voluntary and involuntary.

### What is the Kohnstamm phenomenon?

**Fig. 1:**
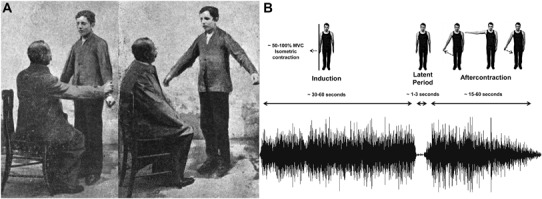
Kohnstamm phenomenon. The first documented image of the Kohnstamm phenomenon (**a**). Dr. Alberto Salmon has one of his patients push outwards against his arms. Upon relaxation, the patient’s arms rise involuntarily due to an aftercontraction of the lateral deltoid muscles (Adapted from Salmon 1916). **b** Modern recording of the Kohnstamm phenomenon showing the basic kinematics, average duration, and a typical EMG trace from the right lateral deltoid muscle

The Kohnstamm phenomenon (Fig. 1.), as originally described, refers to the observation that if one pushes hard outward against a fixed surface with the back of the hand for approximately 30 s and then ceases, an abduction of the arm will occur, accompanied by a feeling that the movement is involuntary and the arm lighter than usual (Kohnstamm 1915; Salmon 1915). When pre-screening is not used, the Kohnstamm phenomenon is reported in about 75% of healthy participants (Adamson and McDonagh 2004; Duclos et al. 2007; Hagbarth and Nordin 1998; Ivanenko et al. 2006). It is not known why some individuals do not display the effect, although general anxiety towards the experimental environment is likely a factor (Craske and Craske 1985). Researchers have noted large individual differences in how easily the aftercontraction can be elicited, and when it is, differences in movement speed and amplitude (Adamson and McDonagh 2004; Kohnstamm 1915; Salmon 1916, 1925). Early work claimed that the Kohnstamm phenomenon displays uniformity across sessions in healthy individuals (Allen 1937), though this has not been verified statistically. The degree variability of the Kohnstamm aftercontraction appears to be consistent with the variability seen in other involuntary movements, such as the tendon jerk reflex (Dick 2003).

While most studies utilise the deltoid muscle (Adamson and McDonagh 2004; Fessard and Tournay 1949; Kohnstamm 1915; Pinkhof 1922; Salmon 1915, 1916), it has always been known that the Kohnstamm phenomenon can be easily demonstrated in many muscles including flexors and extensors of the arm, wrist, ankle, knee, hip, and also the neck muscles (Allen and O’Donoghue 1927; Csiky 1915; Forbes et al. 1926). Indeed, it has been suggested that an aftercontraction can be elicited from any skeletal muscle providing a suitable induction exists (Forbes et al. 1926) and early work documented the aftercontractions in 20 different muscles within the same individual (Matthaei 1924a). However, it was also reported that the Kohnstamm phenomenon is hardest to produce in the muscles of the hand (Matthaei 1924a). Recently, it has been found that aftercontractions emerge more clearly in proximal joint muscles compared to the muscles of distal parts of the limb (Gregory et al. 1988; Gurfinkel et al. 1989). Traditionally, the Kohnstamm phenomenon is studied in the context of a single muscle. Co-contraction of antagonistic muscles such as the biceps and triceps does not produce any aftercontraction (Gilhodes et al. 1992). However, with specific complex movements of the axial muscles, aftercontraction activity is found simultaneously in antagonistic muscles (Ghafouri et al. 1998). Pushing the legs together for extended periods of time can produce involuntary air stepping (Selionov et al. 2013, 2009), demonstrating that complex muscle synergies can be recruited.

In all previous studies, the aftercontraction is elicited via an isometric muscle contraction. This can be achieved by pushing against a solid surface (Kohnstamm 1915) or holding a fixed amount of weight stationary out from the body (e.g., Sapirstein et al. 1937). Even small amounts of force, requiring just 10% of the muscle’s maximum voluntary contraction (MVC), maintained for 10 s, are adequate in some individuals (Allen and O’Donoghue 1927). However, to induce a robust effect across participants, most paradigms involve 50–100% MVC for durations of 30–60 s. It is possible to generate the effect with the muscle at a variety of lengths during the induction (Forbes et al. 1926; Hagbarth and Nordin 1998).

After cessation of the voluntary contraction, there is a latent period. The muscle is not active and the limb is stationary (Gurfinkel et al. 1989; Kozhina et al. 1996). The duration of this period varies across participants, but on average lasts 1–3 s (Csiky 1915; Kozhina et al. 1996; Meigal et al. 1996; Parkinson and McDonagh 2006; Pinkhof 1922; Sapirstein et al. 1937). Typically, participants are instructed to relax to trigger the aftercontraction (Sapirstein et al. 1937; Mathis et al. 1996; Ghafouri et al. 1998). However, it is unknown what signals are necessary to trigger the aftercontraction beyond the cessation of the voluntary contraction. Instruction to relax may result in smaller aftercontractions relative to maintaining normal posture (Hick 1953). However, this observation has not been statistically verified.

The aftercontraction phase of the Kohnstamm phenomenon causes a movement of the limb in the direction of the induction force. In the deltoid, it is routinely reported that in many individuals the arm abducts to the maximum 90° (Adamson and McDonagh 2004; Kohnstamm 1915; Salmon 1916). There is high variability across protocols, but typically, the aftercontraction duration is in the range of 10–60 s (Sapirstein et al. 1937; Gurfinkel et al. 1989; Parkinson et al. 2009), though in one experiment, postural effects were detected for up to 14 min (Duclos et al. 2004). The end of the aftercontraction is poorly defined. With some participants (Matthaei 1924b; Sapirstein et al. 1937) or protocols (Craske and Craske 1985; Forbes et al. 1926), it naturally takes on an oscillatory character. However, in most cases, the arm is brought down from a statically abducted position either by instruction or by the voluntary decision to adopt a new posture. Subjective feeling of lightness may be the best way to gauge the true duration of the aftercontraction (Cratty and Duffy 1969).

### Why study the Kohnstamm phenomenon?

The Kohnstamm phenomenon has been reported in the literature for 100 years. It has likely been known about for much longer (Pereira 1925a) and may be considered a folk illusion (Barker and Rice 2012). General interest in the phenomenon is due to the ease with which the effect can be demonstrated, the accompanying strange sensation, the surprised reaction it evokes in those experiencing it for the first time, and the associated pleasure that comes from both its performance and the passing of ‘secret’ knowledge in a social context (Barker and Rice 2012). However, the Kohnstamm phenomenon is not merely a parlour trick. Early researchers understood the physiological and psychological insights that could be gained from its study. It was central to resolving a long-standing debate about the possibility of muscle contractions without action currents (Forbes et al. 1926; Pereira 1925a; Pinkhof 1922; Salmon 1925; Salomonson 1921; Schwartz 1924; Schwartz and Meyer 1921). After years of sporadic study, scientific interest in the Kohnstamm phenomenon began to increase from the late 1980s to the present day. However, many questions remain regarding its cognitive control. Advances in the understanding of motor control (Bizzi et al. 1984; Marsden et al. 1976a) and the neurocognitive basis of the sense of agency (Blakemore and Frith 2003; Haggard 2008; Shergill et al. 2003; Wolpert and Kawato 1998) mean that there is now a strong theoretical context in which to interpret findings from Kohnstamm experiments. The phenomenon’s status as something of an isolated oddity should not prevent rigorous study. Researchers have long drawn the analogy with visual illusions (Fessard and Tournay 1949; Salmon 1916, 1925), themselves once considered just games, but now recognised as a key source of knowledge about the mechanisms of visual perception. Similarly, the Kohnstamm phenomenon may provide important insights into the fundamental nature of voluntary and involuntary movement control.

Much research has been conducted to try and isolate the involuntary mechanisms of low-level motor control, without the normal overlay of voluntary control. Perturbation studies have focused on responses to unloading the muscle during tasks in which the participant is instructed not to intervene to counteract a perturbation (Archambault et al. 2005; Raptis et al. 2010). Imperceptible perturbations have also been used to bypass voluntary responses to perturbations (Hore et al. 1990). In the case of the Kohnstamm phenomenon, the involuntary processes are amplified and prolonged, allowing the mechanisms to be studied isolated from confounding voluntary interventions.

Isolating the motor commands of other involuntary reflexes, and determining how they contribute to action awareness is difficult because of their rapid onset, short duration, and close interaction with afferent signals (Ghosh and Haggard 2014). The Kohnstamm phenomenon does not suffer from this problem. It is the speed of a slow voluntary movement, meaning that it can be perturbed, and the physiological consequences recorded. The quality of being physically indistinguishable from a voluntary movement, yet subjectively entirely different, makes the Kohnstamm phenomenon an attractive tool to study how these two components of movement are linked. The results of such experiments will elucidate both voluntary and involuntary movement. They may also help to explain where the Kohnstamm phenomenon fits within the range of reflexive, postural, and voluntary motor control. Furthermore, by contrasting voluntary motor control and Kohnstamm movements, important questions about the inhibition of existing movements can be addressed.

### Previous literature

The Kohnstamm phenomenon has also been referred to as the *Katatonusversuch* (Kohnstamm 1915), *after movement* (Csiky 1915), *residual contraction* (Pinkhof 1922), *Salmon-Kohnstamm phenomenon* (Henriques and Lindhard 1921), *automatic movement* (Salmon 1925), *automatic contraction* (Pereira 1925a), *involuntary contraction* (Forbes et al. 1926), *post-contraction* (Allen 1937), and *aftercontraction* (Sapirstein et al. 1937). The literature for the following review was obtained by searching Pubmed and Web of Science using the above search terms. Once all listed studies had been found, additional papers were located by examining the reference lists of all papers. For the purposes of clarity, in this review, the term Kohnstamm phenomenon will be used to refer to the entire effect, while individual stages will be referred to as Induction, Latent period and Aftercontraction. Papers are only included in the table if they are peer reviewed, present original research data, and focus on involuntary aftercontraction (Table 1).

**Table 1:** Chronological summary of research on the Kohnstamm phenomenon

References	Measurement Techniques used	Group size	% Showing AC	Muscles investigated	Induction method	Induction strength	Induction duration	Latent period	Size of AC	Duration of AC	Subjective reports	Key findings
Salmon (1915)	Observation only	No report	No report	Lateral deltoid, bicep, thigh, anterior flexion of trunk, neck extensors	Push hard outwards against experimenters arms or hard surface	No report	No report	No report	No report	No report	Lightness, surprise	1. First report of the AC, which is found in most participants. AC size not strongly dependent on induction strength/duration 2. Easier to elicit in emotionally reactive people 3. AC is stronger in patients with hysteria, absent in schizophrenia, more pronounced in Parkinsons disease, present in Tabes Dorsalis, absent in hemiplegia
Kohnstamm (1915)	Faradic stimulation	No report	No report	Lateral deltoid and leg muscles (no specific details)	Pressing backs of hands against wall with high tension	No report	5–60 s	No report	Up to 120°	No report	Mysterious force, strange, flying	1. Independent discovery of phenomenon. Size of AC depends on the individual and duration of push 2. Faradic stimulation does not produce AC 3. Diminished in cases of Tabes Dorsalis, lacking in people with negativistic personality type, very strong in hypnotised people
Rothmann (1915)	Observation only	No report	No report	Lateral deltoid, pectoralis, wrist extensors, neck muscles	Pressing backs of hands against wall with high tension	No report	5–60 s	No report	No report	No report	Surprise, involuntary, automatic	1. AC restricted to the extensor muscles 2. Found in Tabes Dorsalis, absent in Hemiplegia, absent in patient with Cerebellar damage
Csiky (1915)	Observation only	No report	No report	Lateral deltoid, extensors and flexors of arms and legs	Pressing backs of hands against wall with high tension	No report	30–60 s	2–3 s	No report	12–15 s	Strange feeling, involuntary	1. First to time and define separate phases of Kohnstamm phenomenon (induction, latent period, AC) 2. AC found in both flexor and extensor muscles 3. AC found in some participants after 1 min of intense faradic stimulation
Salmon (1916)	Observation only	No report	No report	Lateral deltoid, knee, arm and neck extensors	Push hard outwards against experimenters arms or hard surface	No report	No report	No report	No report	No report	Lightness, flying	1. AC more common in emotional people. AC is stronger in patients with hysteria, absent in dementia, more pronounced in Parkinsons disease, present in Tabes Dorsalis, absent in hemiplegia 2. No clear relationships between tendon reflex strength and AC across participants
Salomonson (1921)	EMG (string galvanometer)	No report	No report	Lateral deltoid, hand extensors	Isometric contraction of deltoid against rigid surface	max effort	60 s	No report	No report	1–10 s	Arm drawn upwards without, or even against will	1. AC less pronounced in old and apathetic or subjects with early dementia 2. No electrical activity in muscle detected during AC
Danielopolu et al. (1921)	Kinematics (no methodology), injection of caffeine to muscle	No report	No report	Lateral deltoid, biceps, back, neck trunk and leg muscles	Hold heavy weight or push hard	No report	10–15 s	No report	No report	No report	No report	1. AC exists for all voluntary muscles, contraction must be isometric 2. AC highly diminished with repeated inductions (fatigue) 3. Absent AC (deltoid and bicep) in 1 patient with myasthenia gravis, 4 patients with cachexia, but occurred after injection of caffeine
Henriques and Lindhard (1921)	EMG (string galvanometer), faradization of muscle	No report	No report	Lateral deltoid	Pushing against solid surface, leaning with body weight	No report	No report	No report	> 45°	No report	No report	1. Muscle activity at all stages of the Kohnstamm phenomenon (one trace shown, not clear) 2. Leaning with body weight (supposedly no contraction) produced 45° AC. Not present if a cushion used 3. Faradization (1 min) produced small AC
Schwartz and Meyer (1921)	EMG (string galvanometer; no traces shown)	No report	No report	Lateral deltoid	Push against solid surface	Max effort	10–12 s	No report	90°	No report	Surprise, foreign force independent of will	1. Electrical activity in muscle present throughout AC (even when arm stationary at 90°) 2. Similar to activity seen during voluntary action
Pinkhof (1921)	EMG (string galvanometer), kinematics (air tyre surrounding body)	4	No report	Lateral deltoid, bicep, wrist extensors	Push against solid object or hold weight	5 kg	60 s	2 s	No report	Up to 30 s	Like passive movement, flying, weightless, like in water, slight pressure on underside of arm	1. Action currents present during AC for biceps and deltoid, all cases (20 cases from 4 participants) 2. Action currents during AC same intensity and frequency as those of voluntary movements 3. Muscle is silent during latent period (1–2 s)
Pinkhof (1922)	EMG (string galvanometer), kinematics (air tyre surrounding body), electrical stimulation	4	No report	Lateral deltoid, bicep, wrist extensors	Push against solid object or hold weight	5 kg	60 s	2 s	No report	Up to 30 s	Like passive movement, flying, weightless, like in water, slight pressure on underside of arm	1. Reflexes (from electrical stimulation) produced after inductions were similar to those during voluntary contraction 2. Re-reported the results of earlier paper (Pinkhof 1921)
Hazelhoff and Wiersma (1922)	Kinematics **(**Kymograph)	No report	No report	Lateral deltoid	Push against solid surface	No report	No report	No report	No report	No report	Beyond control, involuntary, lightness	1. Imagining movement during Kohnstamm phenomenon produced bigger AC, while distracting attention away from arm stopped AC occurring
Matthaei (1924a)	Spring to measure weight of arm during AC, Faradization	>40	100%	Deltoid, biceps, triceps, hand extensors, quadriceps, psoas, gluteus, hamstrings, hip	Pushing outward on padded surface, weights for other muscles	Up to 5 kg	10–120 s	<1–10 s	Up to 90°	30–60 s	Lightness, passivity, pulled upwards, moves by itself, flight, like a dream. Heaviness at end	1. AC can be induced in any skeletal muscle, rarely in the hand. AC manifest in direction of contraction of muscle, not direction of force 2. Size of AC (distance moved by arm) depended on intensity/duration of induction 3. Alcohol ingestion increases AC size, injecting novocaine in shoulder removes subjective feeling of lightness, but AC unaffected
Matthaei (1924b)	Early form of strain gauge	28	No report	Biceps	Holding suspended weight with arm bent	0.5–5 kg	5–120 s	No report	No report	No report	Lightness	1. Found a logarithmic relationship between induction intensity and size of subjective force overestimation, indicated via voluntary movement of other arm 2. Magnitude of error does not depend on the voluntary hand
Pereira (1925a)	EMG (string galvanometer; cathode amplification)	No report	No report	Lateral deltoid	Hard push against wall	No report	60 s	No report	~90°	No report	No report	1. Electrical muscle activity not detected when arm reached max position during AC and was stationary. Seen only during movement. Obstruction and voluntary inhibition caused action currents to stop, but muscle was still contracting 2. Rapid voluntary contraction, immediately after induction prevented AC
Salmon (1925)	Observation, Faradic stimulation	No report	No report	Lateral deltoid	Resisting the force exerted on the arms by experimenter	No report	No report	No report	No report	No report	Feeling of automaticity, limb lighter, flying	1. AC more pronounced in emotional subjects, subjects with hysteria and subjects gifted with a very vivid imagination (sometimes produced by just mental imagery) 2. Faradic stimulation produced only very weak AC 3. Decreased AC in 2 patients with Tabes Dorsalis, decreased AC on affected side in 2 patients with hemiplegia
Verzár and Kovács (1925)	EMG (string galvanometer; steel needle electrodes)	15	93.33%	Biceps	Hold weight with bent arm (90° angle relative to the upper arm)	5 kg	60–90 s	No report	Up to 120°	No report	No report	1. Action currents during AC with 10–20% fewer waves per second than during voluntary movement (no way to exactly match velocity) 2. Muscle cooling (ice pack 15 min) produced ~20% reduction in waves/s during AC and voluntary movement
Bellincioni (1926)	Kinematics (kymograph)	No report	No report	Lateral deltoid, anterior deltoid, knee extensors	Push hard outwards against solid surface (seated and standing)	No report	No report	No report	Up to 90°	~10 s	Lightness	1. Extension of head, trunk and non-moving arm away from moving arm increased AC size, while extension towards it had opposite effect 2. Rotating chair during induction caused AC to deviate in direction of previous rotation by 20–30° 3. Short period of hyperpnea increased the size of the AC, long periods cause partial or total inhibition
Forbes et al. (1926)	EMG (string galvanometer), Kinematics (kymograph)	7	86%	Lateral deltoid, biceps, pectoralis, triceps, wrist flexors, hip, knee, neck	Seated, push outwards	~ 100% MVC (effort)	20–25 s (also 60 s)	No report	No report	Up to 25 s	Surprise	1. EMG signal present throughout AC, similar to matched voluntary movements 2. Obstruction of arm during latent period abolished AC, but obstruction during AC did not reduce muscle activity (arm held in place at obstacle) 3. Inhibition of arm possible without use of antagonist muscle, easier at start of movement
Allen and O’Donoghue (1927)	Kinematics (protractor)	4	No report	Lateral Deltoid and leg muscles	Wire and pulleys, arm away from body, standing	0.55–4.55 kg	10 s	No report	Up to 100°	No report	Lightness, rise of its own accord, no volition	1. Size of AC increases (logarithmically) with induction strength at fixed duration 2. Fatigue with repeated inductions. Augmentation if a 20 min rest was included 3. Other arm fatigue causes reduction in AC, and then augmentation with rest
Laignel-Lavastine et al. (1927)	Torque device for measuring induction force (no details)	No report	No report	Lateral deltoid	Push against solid surface	4 kg	120 s	0–5 s	45 to 120°	9–45 s	No report	1. AC Abolished in general paralysis caused by syphilis (4 cases), multiple sclerosis (2 cases), early dementia (2 cases) and paranoid dementia (1 case), very decreased for the affected side of hemiplegic patients 2. Very extended AC duration of in Parkinson’s disease (10 cases), melancholia (3 cases), myxedema (2 cases), psychiatric patients (hysteria, phobia, schizophrenics, addicts)
Salmon (1929)	Observation only	No report	100%	Lateral deltoid, bicep (arm flexor), knee extensors (quadriceps), neck extensors	Push hard against solid surface	No report	20–30 s	1–2 s	No report	No report	Feeling that the arm is lighter than normal, flies	1. More pronounced AC in Hysteria patients and patients with Parkinsons or morphine addiction 2. Reduced in Hemiplegia, Early dementia, Tabes Dorsalis 3. Latency increases with longer inductions
Sapirstein, et al. (1936)	Kinematics (Kymograph), administration of drugs	60	No report	Hip flexion	Supporting suspended weight	6 kg	15 s	No report	No report	No report	No report	1. Leg AC markedly reduced after 2 gm sodium bromide (often abolished, despite knee jerk being normal) 2. Caffeine (0.15 g) found to increase size of AC. Very effective at offsetting suppression by sodium bromide 3. Other drugs (chloral hydrate, strychnine and barbital) found to have lesser effect
Sapirstein et al. (1937)	Kinematics (Kymograph), administration of drugs	No report	No report	Hip flexion	Supporting suspended weight	1–6 kg	10–25 s	Up to 3 s	No report	3–40 s	No report	1. Increased strength and duration of induction produces bigger AC 2. Dorsiflexion of the foot increased the size of hip AC. Abducting ipsilateral arm with 2 kg weight caused increase in leg AC. Contralateral arm usually produced decrease, but sometimes produced an increase 3. AC can be prevented by exerting a voluntary force in the other direction at the point of relaxation. If movement is restrained by experimenter at relaxation AC is delayed
Allen (1937)	Kinematics (protractor)	5	No report	Lateral deltoid and leg muscles	Wire and pulleys, arm away from body, standing	0.55–4.55 kg	10–15 s	No report	Up to 75°	No report	Involuntary, detachment, lightness, floating, weight loss	1. Bigger and longer induction increases AC size 2. Fatigue reduces AC size 3. Right leg contractions during right arm induction, reduced size of right arm AC
Holway et al. (1937)	Kinematics (protractor), adjustable weight balance	3	No report	Lateral deltoid	Push outwards against weighted balance	0.02–6.4 kg	15 s	No report	Up to 122.8°	No report	No report	1. Size of AC found to be a power function of force during induction (wide range of forces)
Sapirstein et al. (1938)	Kinematics (Kymograph), administration of drugs	>20	No report	Hip flexion	Supporting suspended weight	3–6 kg	15 s	No report	No report	No report	No report	1. Normal AC found in 10 Tabes Dorsalis patients, small AC found in 2. No correlation between severity of condition and size of AC 2. Prolonged AC in Parkinson’s disease, jerky in single case of cerebellar damage 3. AC reduced in hemiplegia on affected side (spinal reflexes hyper-sensitive)
Wells (1944)	Observation only	No report	No report	Lateral deltoid, knee extensors	Push outwards against solid surface	No report	60–120 s	No report	No report	No report	No report	1. During bilateral AC, turning head to right, or turning eyes strongly to left, or shining strong light into eyes from left, increases AC of right arm and diminishes or abolishes on the left 2. Forceful downward eye rotation or backward tilting of the head increases AC. Opposite (i.e. upward eye rotation etc.) reduces AC 3. Similar pattern observed in knee extensor muscles
Sapirstein (1948)	Kinematics (Kymograph), administration of drugs	No report	No report	Hip flexion	Supporting suspended weight	3–6 kg	16 s	No report	No report	No report	No report	1. AC is absent in affective psychosis, severe depression, manic depression. AC absent in 3 cases of depression - appeared after electro-shock treatment 2. AC normal in schizophrenia, providing there was no accompanying emotional disturbance 3. Lack of AC linked to anxiety in patients with OCD, phobias and anxious hysteria
Zigler et al. (1948)	Kinematics (Protractor)	4	No report	Lateral deltoid	Pull on cord holding suspended weight	0.8–3.2 kg	7.5–30 s	No report	No report	No report	No report	1. Across a range of strength and durations of inductions, size of AC rapidly increased with successive trials and then gradually decreased with fatigue
Fessard and Tournay (1949)	EMG (single traces, needle electrodes), kinematics (photo-electric instruments)	4	No report	Lateral deltoid, pectoralis	Arm ~20° abducted push outward	No report	5–120 s	2.7–6.3 s	Up to 70°	3.5–37 s	Surprise	1. Duration and amplitude of aftercontraction depends on duration of induction 2. Matched voluntary actions show similar EMG. Voluntary movement on top of AC does not abolish AC. Muscular atrophy patient showed same unusual EMG pattern during AC and voluntary movement 3. Adducting (inhibition) does not abolish the Kohnstamm, there are up to 6 spontaneous recoveries
Paillard (1951)	Kinematics (mechanogram, potentiometric sliders system)	No report	No report	Lateral deltoid	Pushing outward on solid surface	Max effort	5–30 s	No report	up to 80°	No report	No report	1. Bilateral AC was smaller (~25°) than unilateral (~80°) 2. If AC is prevented in one arm at start of bilateral AC, the other arm rises to the normal angle (~80°) 3. Fast voluntary upward movement of right arm causes temporary inhibition of a left AC (stronger if a 2 kg weight held). Final arm angle similar to normal AC, after plateau
Hick (1953)	Spring to measure force	14	No report	Lateral deltoid	Pushing outwards against spring	Up to 3.63 kg	15 s	No report	No report	No report	No report	1. Cognitive distractor task (write name backwards) produced bigger AC effect than baseline 2. Voluntary movements (“produce this force”) could be superimposed on top of AC 3. Instruction to maintain 0 force induced more AC force then instruction to relax after induction
Sapirstein (1960)	Kinematics and EMG (no data shown)	>200	No report	Knee extension, Hip flexion, lateral deltoid	Supporting suspended weight	7.26 kg	20 s	No report	No report	No report	No report	1. Of 200 patients at psychiatric hospital, AC appearance pre-empted improvement, AC loss pre-empted decline in mental health 2. Patients with depression rarely had AC. 17/19 depressed patients had AC only after electro-shock therapy 3. Association between negative emotions and lack of AC. Outward anger did not reduce AC
Cratty and Duffy (1969)	Subjective reporting of effect	39	86%	Lateral deltoid	Standing in constructed doorframe	100% Effort	5–20 s	No report	No report	Mean 14 s	Arm felt lighter than normal	1. Duration of Kohnstamm (defined by self-report of subjective feeling of lightness) was not correlated with strength of other aftereffects (e.g. position errors)
Howard and Anstis (1974)	Moveable trolley to indicate head position with hands	12	No report	Neck	Resisting suspended weight	95 gm	10 min	No report	Up to 24°	No report	No report	1. Pointing accuracy to head position did not differ from baseline during neck AC 2. Pointing accuracy to head position after head turning showed bias to direction of turn (postural persistence)
Craske and Craske (1985)	Kinematics (receiving microphone)	55	No report	Deltoid, triceps, gluteus	Push against solid surface (various postures)	Max effort (exp. 1), moderate (exp. 2 and 3)	30 s	No report	36.35° (median)	median 219.65 s	Surprise, lightness, floating, move of own accord, without decision or intention	1. AC has an oscillatory quality (5.5 median no. cycles) 2. Simultaneous AC in shoulder and forearm produce oscillations of same frequency (16/20). In phase (6/15), rest in 180° or 90° phase 3. Oscillations could be transferred to an un-induced limb by silently naming the limb
Craske and Craske (1986)	Kinematics (receiving microphone)	52	No report	Deltoid	Push against solid surface (various postures)	50% MVC	30 s	No report	Exp. 1: 9.9°; Exp3: 34.15°	No report	No report	1. Oscillatory AC can be transferred from inducted arm to other arm by naming the limb 2. Oscillations in right and left arm interact when inductions are in different planes 3. AC (34.15°) can be induced by motor imagery
Gurfinkel et al. (1989)	EMG, kinematics (mechanogram), vibration, electric stimulation	7	No report	Calf, quadriceps, hand extensors, lateral deltoid, trunk	Lift weights against gravity	2–5 kg	30–60 s	No report	>30°	40–50 s	Lightness	1. Induction with distal muscle sometimes switched to proximal muscle AC. Also is produced by muscle vibration (up to 20 min later) 2. Deltoid AC larger in standing versus sitting subjects (even larger if standing on toes) 3. Electrical stimulation failed to produce AC
Gilhodes et al. (1992)	EMG, kinematics, vibration, electronically controlled eye mask	14	71.43%	Biceps and triceps	Seated, push against static restraint (arm bent at 95°)	4–5 kg	30 s	No report	No report	>60 s	No report	4. In darkness eyes opening and closing had no effect, but in diffuse light opening and closing correlated with switch back and forth between muscles (bicep/triceps) 5. Muscle switching occurred for both bicep and triceps inductions. Not if co-contracted 6. Same effect achieved via vibration
Mathis et al. (1996)	EMG, kinematics (potentiometer), TMS, vibration	7	No report	Lateral deltoid	Seated, arm abducted (10–20°) push outwards against counter weight	4–6 kg	40–60 s	No report	20–72°	No report	No report	1. MEP size correlated with background EMG level for AC and matched voluntary movements. MEP amplitude, gain, latency and dynamics did not differ. Similar results for vibration induced movement 2. Found bigger MEPs for rising EMG (i.e. muscle shortening) compared to falling EMG in 20% of Vol trials and 30% of AC trials
Kozhina et al. (1996)	Single motor unit recording (intramuscular needle electrodes), EMG, kinematics (goniometer)	4	No report	Lateral deltoid, triceps and anterior tibialis	Pulling up on handle or pushing out against elastic band	50–70% MVC	40 s	1.4 s	30–40°	~10 s	No report	1. Mean firing rate of motor units significantly lower during AC (12 pps) compared to matched voluntary movements (14 pps) 2. Other properties (e.g. spike amplitude) did not differ 3. Firing rate increased with movement. Very low firing rate if movement prevented before AC developed
Meigal et al. (1996)	EMG, heating and cooling of entire body	6	No report	Biceps and triceps	Flexion of elbow against sold plate	70% MVC	60 s	2–3 s	No report	1–6 min	No report	1. Cold air exposure (+ 5 °C), increased EMG (%MVC) during AC, relative to room temperature (+ 22 °C). Hot air exposure (+ 75 °C) decreased AC EMG and duration 2. AC sometimes spontaneously transferred from biceps to triceps
Hagbarth and Nordin (1998)	EMG, kinematics, muscle cooling/heating, vibration	14	71.43%	Lateral deltoid	Pushing upwards against solid surface, arms at 90°	0–100% max effort	~20 s	No report	~10°	~10 s	Lightness, involuntary	1. Omission of steps of muscle conditioning procedure (from animal literature to maximise post-contraction afferent discharge) reduced size of AC 2. Warming muscle produced significant decrease in AC size. Cooling produced trend towards increase in AC size 3. AC from vibration same as from contraction
Ghafouri et al. (1998)	Kinematics (scapula: 3D optical motion analysis), EMG	10	60%	Trapezius pars descendens and latissimus dorsalis	Produce isometric contraction against weight attached in shoulder bag	8 kg	360 s	No report	No report	50–60 s	No report	1. Greater EMG during standing than sitting AC. Different activity in the two muscles 2. Different direction of spiral unrolling motion of scapula in standing (clockwise) and sitting (anticlockwise) 3. Opening eyes after induction triggered AC switch from traps to lats in standing, but not sitting condition
Brice and McDonagh (2001)	Force, Kinematics (goniometer)	6	No report	Lateral deltoid and leg muscles	Arm 30° abducted, push outward, standing	20–100% MVC	15–75 s	No report	Up to 92°	No report	No report	1. Threshold induction duration is required to produce AC. Beyond this, magnitude of AC proportional to force generated during induction
Lemon et al. (2003)	EMG, strain gauge, tilt table	9	No report	Lateral deltoid	Pushing outwards against strain gauge	60% MVC	60 s	No report	No report	No report	No report	1. Mean AC EMG decreased almost linearly from 46.6% MVC when upright to 12.7% MVC when supine
Adamson and McDonagh 2004)	Strain gauge, Kinematics (goniometer), EMG, cuffing wrist	9	~70%	Lateral deltoid	Arm 15–20° abducted push outward, standing	100% effort, dropped to 60% by end	60 s	1–5 s	Up to 70°	~60 s	No report	1. AC EMG (%MVC), when arm obstructed, is linearly dependent on joint angle 2. EMG on downward adduction is linearly dependent on position, but lower 3. Changes in EMG not dependent on cutaneous input
Duclos et al. (2004)	Force, centre of pressure recordings, electrical stimulation	14	No report	Neck muscles (splenius, trapezius, obliques)	Pushing head against differently positioned pads	50% MVC	30 s	No report	No report	Up to 14 min	No report	1. Immediate, long lasting whole body leaning, specific to muscle contracted 2. Did not occur after electrical stimulation of muscle
Ivanenko et al. (2006)	Kinematics (Motion tracking cameras), strain gauge for induction	21	75%	Trunk	Resist a rotational torque applied at the pelvis	40 N m (rotational torque)	30 s	No report	~5°	Up to 40 s	No report	1. Trunk AC produced curved deviations (10%) in voluntary walking in the direction of induction contraction 2. Did not occur when stepping on the spot
Parkinson and McDonagh (2006)	Kinematics (goniometer), EMG, pivot lever arm with moveable counter-weight	10	No report	Anterior deltoid	Shoulder flexion (40°) seated, pushing upwards on solid surface	60% MVC	60 s	2–5 s	Up to 90°	~60 s	Lightness, movement due to external force	1. AC EMG (% of induction) linearly decreased at every arm angle with increased assistive counter-weight (decreased load: 100–0%)
Duclos et al. (2007)	fMRI, EMG, vibration	11	No report	Wrist extensors	Push upwards (wrist 10° extended) against solid surface, supine	50% MVC	30 s	No report	Up to 30°	50 s	No report	1. AC associated with activity in primary sensory and motor cortices, premotor cortex, anterior and posterior cingulate, parietal regions, insula and vermis of cerebellum 2. Supplementary motor area (BA6) active during voluntary movement, not AC. Cerebellar vermis more active during AC 3. Activation during AC similar to during TVR
Parkinson et al. (2009)	fMRI, kinematics, EMG (outside scanner)	11	No report	Anterior deltoid	Shoulder flexion, pulling upwards on rope attached to body, lying supine	100% MVC (effort)	60 s	1–2 s	11.54 cm disp	~30 s	No report	1. Widespread cortical and sub-cortical activation during AC (motor cortex, pre- central gyrus, superior parietal, caudate, thalamus, cerebellum) 2. Greater activity in supplementary motor area and anterior cingulate during AC than voluntary movement 3. Greater activity in putamen during voluntary movement than during AC
Selionov et al. (2009)	EMG, kinematics (potentiometers, elastic chord to measure force)	18	88.89%	Hip flexor and leg extensor muscles	Supine, legs supported. One leg pushing forward, the other back against each other	50% MVC	30 s	No report	No report	5–60 s	No report	1. Observed rhythmic air stepping (forward motion) activity in both legs for about 15 s after induction 2. EMG showed AC in multiple muscles 3. Maximal frequency and amplitude of the hip and knee joint movements occurred after 3–7 cycles
Meigal and Pis’mennyi (2009)	EMG, heating and cooling of entire body	102	82%	Lateral deltoid and biceps	Pushing outwards against belt and flexion of elbow against table underside	50% MVC	60 s	No report	No report	mean = 60 s, max > 5 min	No report	1. Body heating reduced the duration of the biceps AC. Cooling increased biceps AC EMG (% MVC) 2. Hot air exposure produced a trend towards increased AC EMG (%MVC) in deltoid. Cooling had no effect 3. 76% of participants had long AC (arm held horizontal), 10% had rapid AC (arm rose and fell in 30 s), 8% showed oscillatory AC, 8% no AC
Selionov et al. (2013)	EMG, kinematics (potentiometers, elastic chord to measure force)	47 (22 controls, 25 patients)	50% of controls, 4% of patients	Hip flexor and leg extensor muscles	Supine, legs supported. One leg pushing forward, the other back against each other	50% MVC	30 s	No report	No report	5–60 s	No report	1. AC air stepping found in 50% of controls, but only 1/25 Parkinsons patients (did not appear after dopaminergic treatment)
Ghosh et al. (2014)	EMG, kinematics (LEDs and 60fps camera), TMS (single pulse)	39	~70%	Lateral deltoid	Push outwards against solid surface, arms slightly abducted (15°)	40–60% MVC	40–60 s	No report	Up to 90°	No report	Sense of resistance when voluntarily adducting during AC	1. TMS to primary motor cortex during AC induces silent period in agonist muscle. Silent period has same latency and duration as during voluntary movement 2. Voluntarily inhibition of AC; bring arm down, then additional ACs without use of antagonist 3. Voluntary inhibition (adduction) associated with stronger subjective feeling of resistance than when no AC present
Brun et al. (2015)	EMG, kinematics, manipulandum, strain gauge, vibration	21	~70%	Biceps	Pulling upwards on handle	40% MVC	35 s	1–2 s	~30°	~10 s	No report	1. Velocity of bicep AC adjusts towards velocity of a passive movement of other arm 2. Velocity of bicep AC adjusts towards increasing velocity of a simulated movement of other arm (increasing vibration frequency: 25-75Hz)
De Havas et al. (2015)	EMG, kinematics (LEDs and 60fps camera), strain gauge	39	84.62%	Lateral deltoid	Push outwards against solid surface, standing	~70% MVC	30 s	No report	mean = 39.83°	No report	Involuntary, lacked agency, automatic, lightness	1. Obstruction of AC caused EMG to plateau, removal caused immediate increase, arm reached same final angle as if no obstacle present 2. During bilateral AC, obstruction of one arm had no effect on unobstructed arm EMG 3. Comparison to matched voluntary movements revealed preserved stretch response when AC arm first contacts obstacle, and overestimation of perceived contact force
Brun and Guerraz (2015)	Kinematics, manipulandum, strain gauge, vibration	40	72.5%	Biceps	Pulling upwards on handle	40%	30 s	No report	~45°	~10 s	No report	1. Passive displacement of one arm slowed AC in other arm 2. Effect abolished when passive arm had proprioceptive masking (agonist and antagonist vibration) 3. Effect facilitated by vision of the passively moved arm, mirrored to appear in Kohnstamm arm location
Solopova et al. (2016)	EMG, kinematics (potentiometers, elastic chord to measure force). Muscle vibration	22	75% (*n* = 5 pre-screen)	Posterior and anterior deltoid, biceps, triceps, flexor carpi radialis, rectus femoris, biceps femoris, lateral gastrocnemius, tibialis anterior	Supine, body supported. One arm pushing forward, the other back against each other. Tested legs in same way	~50% MVC	~30 s	No report	No report	8–60 s	No report	1. Induction produced involuntary alternating movements of both arms. Triggered rhythmic leg movements in 6/15 participants 2. Arm oscillation amplitude increased then steady, but frequency was constant during involuntary movement and similar to that induced by muscle vibration 3. Movement accompanied by EMG in multiple muscles, ~20–30% higher if muscle used in isometric induction
De Havas et al. (2016)	EMG, kinematics (LEDs and camera), strain gauge	21	75%	Lateral deltoid	Push outwards against solid surface, standing	~70% MVC	30 s	2.82 s	Mean = 47.25°	Mean time to reach max angle = 20.81 s	Involuntary, lacked agency, automatic, lightness, interesting, pleasant, dreamlike, smooth	1. AC can be inhibited (arm stationary, partially abducted) without antagonist muscle. Involuntary movement starts when inhibition relinquished, reaches same final angle as if not inhibited 2. Inhibition may involve negative motor command. Sums with output of putative Kohnstamm generator (generator not affected by inhibition) 3. AC force overestimated, relative to voluntary contractions with similar EMG

*AC* aftercontraction

### Summary of table

The table identifies 62 original research papers. The most prolific decade for research was the 1920s (17 papers), and there was then a steady decline until the 1980s when interest began to increase. The table includes 41 papers written in English, 10 in French, 7 in German, 2 in Italian, and 2 in Dutch. The most prolific authors are Victor Gurfinkel (8 papers: 1989–2016), Martin McDonagh (5 papers: 2001–2009), Milton Sapirstein (5 papers: 1936–1960), and Albert Salmon (4 papers: 1915–1929). Research was published from the USA (11 papers), France (10), UK (9), Italy (8), Germany (5), Canada (5), Russia (5), Netherlands (4), Hungary (2), Denmark (1), Switzerland (1), and Sweden (1).

Numbers of participants were not typically reported prior to the 1950s. It is difficult to estimate the mean number of participants included in subsequent studies because some experiments used pre-screening, while others did not. Likewise, the prevalence of the aftercontraction is skewed by pre-screening, but appears to be 70–80% of healthy participants. Kinematic recording was used in 40 experiments, EMG in 31 experiments, fMRI in 2 experiments, and TMS in 2 experiments. The most commonly studied muscle is the deltoid, which was used in 46/62 papers. A variety of methods have been used to induce the aftercontraction, but they all involve isometric contractions and an attempt to maintain a constant force, either against gravity (holding weight) or a fixed surface (pushing). A standard Kohnstamm induction is 40–100% MVC for 20–60 s. Only two studies (De Havas et al. 2016; Kozhina et al. 1996) appear to have reported accurate mean data for the latent period between the end of induction and the onset of aftercontraction. Others report a range with the general consensus being that the mean is 1–3 s. Little can be concluded about the size and duration of the aftercontraction owing to the wide range of methodologies used and muscles studied. Reports of the mean size and duration of the aftercontraction are surprisingly rare, perhaps because many studies used more than one induction protocol. However, it can be noted that aftercontractions of the deltoid can induce involuntary movements of up to 90°, using a variety of inductions. The typical duration of the aftercontraction appears to be 10–60 s. The percentage of this time involving a moving versus stationary limb varies considerably across individuals. Key findings are discussed in the following.

## Research themes

### What is happening at the muscle during the Kohnstamm phenomenon?

The muscle itself is the logical starting point for an exploration of the causes of the Kohnstamm phenomenon. Initial work concerned a wholly muscular origin (but see Rothmann 1915; Salmon 1915, 1916). Csiky (1915) was the first to time and formally describe the individual phases of the Kohnstamm phenomenon. He noted a close analogy with the optical afterimage. Both were considered by him to be caused by fatigue of the peripheral apparatus. Supporting this muscular theory, high levels of electrical stimulation of the muscle could apparently induce an aftercontraction (Csiky 1915). However, this was not replicated (Duclos et al. 2004; Gurfinkel et al. 1989; Kohnstamm 1915; Matthaei 1924a) and it is likely that the original finding was due to the participants voluntarily contracting against the direction of the powerful shocks (Zigler 1944). With the availability of the string galvanometer, it became possible to measure innervation of the muscle. Early attempts showed a lack of EMG activity during the aftercontraction (Salomonson 1921), suggesting that muscle tone was maintained without central innervation (Salomonson 1921). Kohnstamm’s (1915) own theory was that the aftercontraction depended on the muscle taking on a new equilibrium point during the ‘hard push’ and then trying to return to that point. He speculated that muscle tone was normally maintained in this local manner and that it was an inhibition of the voluntary movement signal that actually allowed the arm to move. However, this ‘holding back’ of the arm is fundamentally incompatible with the characteristic latent period of 2–3 s (Csiky 1915). Further experiments showed EMG activity during the aftercontraction (Henriques and Lindhard 1921; Pinkhof 1921, 1922; Schwartz and Meyer 1921; Verzár and Kovács 1925). There was a debate as to whether these were products of the movement itself (Pereira 1925a, b) or true central innervation (Salmon 1925), but this was elegantly resolved by showing that they persisted even when the involuntarily rising arm was obstructed (Forbes et al. 1926). Later, modern electromyographic (EMG) recording convincingly showed central motor drive during aftercontraction (Fessard and Tournay 1949), allowing purely muscular theories to be abandoned.

Central innervation does not preclude changes in the muscle from being the origin of the aftercontraction. Such peripheral changes are the basis of the muscle thixotropy hypothesis (Gregory et al. 1988; Hagbarth and Nordin 1998), which remains an influential account of the Kohnstamm phenomenon. Here, the key factor in generating the Kohnstamm phenomenon is changes in the stiffness and slackness of intrafusal muscle fibres. The theory states that a Kohnstamm aftercontraction of the deltoid muscle occurs for the following reasons: (1) under normal conditions when we move our arms, the alternating stretching and shortening movements of largely inactive muscle lead to development of slack in muscle fibres, including intrafusal fibres. As a result, the levels of maintained spindle activity remain low; (2) conversely, the Kohnstamm induction involves static contraction of muscles at short length, resulting in the taking up of slack in the intrafusal fibers; (3) during this voluntary, isometric induction contraction, stable actin and myosin cross bridges form in intrafusal muscle fibers; (4) relaxation causes the arm to be slowly brought back to a longer muscle length; (5) stable cross bridges in intrafusal fibers remain, maintaining them in a state of relative shortness (compared to their state following alternative contraction histories such as if the arm was moving normally); (6) relative shortness in intrafusal muscle fibers causes muscle spindles to be stretched and to send afferent signals; and (7) spindle signalling causes muscular contraction via spinal reflexes. This reflexive response constitutes the aftercontraction.

**Fig. 2:**
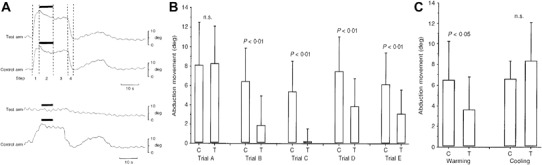
Evidence for muscle thixotropy underlying the Kohnstamm phenomenon. The first panel **a** shows arm movement during the conditioning procedure. Normally, the full conditioning procedure was performed on one arm (control) and a reduced version, with some steps omitted was performed on the other arm (test). However, the *upper panel* here shows single trials when the full procedure was performed for both arms. This consisted of: (1) voluntary arm abduction up against solid surface; (2) forceful, voluntary abductor contraction against solid surface (5–10 s; filled *bar* on graph); (3) relaxation with experimenter holding the arms in place (4–8 s); and (4) experimenter assisted lowering of arms. After step 4, the aftercontraction occurred. The lower panel (**a**) shows a single trial, where performing the induction contraction with the arm partially abducted for the test arm (longer muscle length) leads to an absence of aftercontraction, while an aftercontraction was clearly present for the control arm (short muscle length). The second panel **b** shows the size of aftercontractions after omitting steps from the induction (*C* control arm, *T* test arm). For Trial A, the same conditioning procedure was used on both arms. For trial B, the initial arm abduction was omitted for the test arm, for trial C, the voluntary isometric contraction was omitted for the test arm, for trial D, the experimenter-assisted relaxation period was omitted for the test arm, while for trial E, the test arm was returned rapidly instead of slowly. The third panel **c** shows that warming the test arm significantly reduced the size of the aftercontraction, while cooling produced a trend in the other direction, relative to the control arm (Figure Adapted from Hagbarth and Nordin 1998)

Hagbarth and Nordin (1998) modified a muscular conditioning sequence (used in animals to enhance resting spindle discharge) to act as a Kohnstamm induction for the lateral deltoid muscle (Fig. 2). The sequence involved: (1) participants first holding both arms slightly abducted; (2) actively lifting up their arms against two solid stands (deltoid shortened) and forcefully pressing (max effort) for 5–10 s; (3) relaxing their arms while the experimenter held them up in the fully abducted position (deltoid held short) for 4–8 s; and (4) having their arms passively adducted (slow lengthening of deltoid) by the experimenter to the start position (Fig. 2a). On each trial, the full procedure was performed on one arm, while on the other arm, one of the steps would be systematically omitted. The procedure was found to produce a small aftercontraction with a mean angular displacement of 8°. Omitting any of the steps produced a significant decline in the amount of angular displacement (Fig. 2b), suggesting that the aftercontraction was largest when a procedure was used that maximised the maintenance of shortness and stiffness in the intrafusal muscle fibres. For example, omitting the step that involved passive holding of the muscle at maximum abduction for 4–8 s, purportedly reduced the aftercontraction, because it reduced the gradual formation of stable cross bridges (Fig. 2b; trial B). Replacing the slow, passive adduction with a fast movement purportedly reduced the aftercontraction, because it disrupted the existing stable cross-bridges (Fig. 2b; trial E). The aftercontractions were much smaller than typically seen during a deltoid Kohnstamm (Adamson and McDonagh 2004; Brice and McDonagh 2001; Fessard and Tournay 1949; Laignel-Lavastine et al. 1927; Matthaei 1924a; Paillard 1951; Pereira 1925a; Schwartz and Meyer 1921; Verzár and Kovács 1925). Thus, voluntary movements may have contributed to the effect: for example, knowledge of the complexity of the induction may have set up an expectation of movement size. However, Hagbarth and Nordin (1998) also found that heating the muscle by 3–4 °C significantly decreased aftercontraction size, while cooling by the same amount produced a trend towards an increase (Fig. 2c). This result also fits the thixotropy hypothesis. Muscle temperature may increase (heating) or decrease (cooling) the effects of Brownian motion on the weak physico-chemical bonds that form the actin–myosin cross bridges (Edwards et al. 1972; Lakie et al. 1984, 1986; Sekihara et al. 2007). Indeed, significant whole-body heating and cooling effects on the size of the EMG response during aftercontraction (Meigal et al. 1996) were reported. Muscle cooling was also reported to reduce the frequency of muscle activity during the aftercontraction (Verzár and Kovács 1925). Interestingly, recent evidence suggests the effects of heating and cooling on the Kohnstamm phenomenon may be more complex. Aftercontraction in the biceps was significantly increased by whole body cooling, and tended to decrease with whole body heating (Meigal and Pis’mennyi 2009). Conversely, in the deltoid muscle, whole body cooling had no effect, while heating resulted in a larger aftercontraction.

Whether the muscle itself is the origin of the Kohnstamm phenomenon depends on the spindle discharge being high enough to generate a sufficiently strong and sustained ‘reflex response’. For example, thixotropy models explain the Kohnstamm phenomenon by pointing out that the muscle contraction history can increase spindle sensitivity, through formation of stable cross bridges. In the animal literature, spindle ‘after effects’ are well established (Burke and Gandevia 1995), with numerous studies showing sustained firing following the cessation of a muscle contraction (Brown et al. 1969; Morgan et al. 1984; Gregory et al. 1988, 1990). In the cat, resting discharge of 60% of muscle spindles has been found to be significantly increased for up to 15 min following electrically induced contraction (Hutton et al. 1973). Similar results have been obtained following isometric contraction (Suzuki and Hutton 1976). There is also supporting microneurographic evidence in humans showing spindle after effects (Edin and Vallbo 1988; Macefield et al. 1991). Short periods of isometric contraction of the ankle (5 s) produce 65% increases in spindle firing rates, lasting up to 52 s (Wilson et al. 1995). Other human research has found that fewer than 15% of primary spindles show any post-contraction sensory discharge and that this discharge never exceeds 40 s in duration (Ribot-Ciscar et al. 1998, 1991). However, it should be noted that discrepancies are expected when comparisons are made to the animal literature, owing to differences in physiology and the difficulties of performing microneurography in humans (Burke and Gandevia 1995).

How might spindle after effects produce the Kohnstamm phenomenon? On one account, the isometric voluntary inducing contraction may ‘sensitise’ the muscle spindles (Burke and Gandevia 1995). The resulting increased spindle firing would continuously generate the aftercontraction via spinal and transcortical reflex pathways (Hagbarth and Nordin 1998). However, there is evidence to suggest this account may be incomplete. Following a muscle contraction, increased spindle firing rates are abolished by stretching the muscle (Wilson et al. 1995). Observations involving obstructing the aftercontraction (Forbes et al. 1926), adducting against the aftercontraction (Fessard and Tournay 1949; Ghosh et al. 2014), and tapping the tendon during aftercontraction (Gurfinkel et al. 1989), suggest that introducing stretch to the muscle does not eliminate the Kohnstamm phenomenon. Recent experiments showed that brief (~2 s) obstruction of the arm does not abolish the involuntary aftercontraction and that once the obstacle is removed, the arm rises to the same angle as if no obstacle had been present (De Havas et al. 2015). Furthermore, it was found that obstruction of the involuntary movement by the obstacle produced a stretch response, but that the stretch-induced increase in EMG did not differ in amplitude from that elicited during obstruction of matched voluntary movements. Contrastingly, the muscle thixotropy account predicts that a stretch response would be larger than normal due to the shortness of intrafusal muscle fibers and the resulting increase in spindle gain. The theory also predicts that a perturbation-induced stretch of the muscle should disrupt actin–myosin cross bridges, which should then reduce the strength of the aftercontraction. Neither effect was observed. Finally, the deltoid aftercontraction was observed to be still present after novocaine (20 cc., 1% solution) was injected into the muscle (Matthaei 1924a). The extent of the afferent block was not documented, so interpretation is problematic. However, taken together, the evidence suggests that the Kohnstamm phenomenon is unlikely to be driven solely by the thixotropic state of the muscle (*for a summary of the evidence for and against purely peripheral theories of the Kohnstamm phenomenon* see Table 2).

**Table 2:** Theories of the control principles of the aftercontraction

Theory	Control principle	Evidence for	Evidence against
Purely Peripheral	Aftercontraction driven by high by muscle spindle firing rates, due to alterations in muscle thixotropy caused by induction procedure	1. Microneurographic recordings in humans and animals have shown sustained spindle firing rates following cessation of isometric muscle contractions 2. Conditioning procedures designed to maximise thixotropic changes produced aftercontractions in humans	1. Sustained afferent discharge is small, transient and easily disrupted 2. Stretch reflex in response to hitting obstacles found to be slightly smaller during aftercontractions compared to matched voluntary movements
Purely central persistence of motor command	Ballistic, feedforward control resulting from a persistence of cortical motor activity. Kohnstamm motor command during aftercontraction is not modulated by afferent feedback	1. Early work suggesting that patients with motor cortex lesions had reduced aftercontractions, while partially deafferented patients had preserved aftercontractions 2. Imaging and TMS studies showing cortical involvement in Kohnstamm phenomenon	1. Aftercontraction EMG found to be strongly modulated by afferent signals resulting from hitting obstacles and to be reduced by reductions in the load on the muscle 2. Aftercontraction muscle switched by large changes in visual input
Negative position feedback	Kohnstamm motor command depends on the discrepancy between a central specification of a muscle equilibrium point, and muscle spindle input specifying the disparity between current arm position and the equilibrium value. Equilibrium value may move over time, defining a “virtual trajectory”	1. Aftercontraction EMG level is highly dependent on limb position 2. Aftercontraction overlaps with voluntary movement in terms of central structures recruited. Good evidence for negative position feedback control underlying voluntary movement	Hitting obstacles during aftercontraction does not produce the sustained increase in EMG predicted by negative position feedback control theories
Positive force feedback	Kohnstamm motor command depends on a positive feedback loop between a central excitatory drive and Golgi tendon organ afferent firing rates	Reducing the load on the muscle has been found to reduce aftercontraction EMG across joint angles	1. Difficult to discount negative position feedback control since reductions in muscle load likely involve reductions in spindle firing rates 2. Removal of physical obstruction (muscle unloading) produced increased EMG, rather than decreased

Alternatively, spindle after effects may establish central changes, leading to the aftercontraction being maintained even after spindle firing rates have returned to ‘normal’ levels. This could involve alterations of the plateau properties of spinal motoneurones. The finding that spinal motoneurons demonstrate persistent inward currents, producing sustained firing independent of descending input, is well established in the animal literature (Hounsgaard et al. 1984; Bennett et al. 1998). These plateau properties may be triggered by the kind of large afferent input resulting from post-contraction spindle discharge, establishing sustained and non-linear motor output (Binder et al. 1993). There is increasing evidence for the existence of plateau properties in humans (Heckman et al. 2008; Wilson et al. 2015). Such a mechanism underlying the Kohnstamm phenomenon would account for the sustained, involuntary nature of the aftercontraction and the associated subjective experience. It would also explain why stretching the muscle once the aftercontraction has begun and does not abolish the muscle contraction. However, currently, it is not possible to study the plateau prosperities of spinal motoneurons directly in humans, and no experiments have established a direct link to the Kohnstamm phenomenon.

### What sensory signals reach the brain?

Other, non-muscular afferent signals interacting with the central nervous system may explain the origin of the Kohnstamm phenomenon. Cutaneous signals from the dorsum of the arm during induction were proposed as a cause (Henriques and Lindhard 1921), but can be dismissed due to numerous experiments using suspended weights to elicit the isometric contraction and subsequent aftercontraction (Allen 1937; Allen and O’Donoghue 1927; Ghafouri et al. 1998; Pinkhof 1922; Sapirstein et al. 1937). Afferent signals from the muscle spindles have received more support (Forbes et al. 1926; Matthaei 1924a; Pinkhof 1922; Schwartz 1924; Schwartz and Meyer 1921; Zigler 1944). Theoretically, this afferent signal would drive the aftercontraction by: (a) establishing central adaptations during the induction; (b) altering continuous reflex loops with central regions during the aftercontraction; or (c) a combination of both. Evidence for the role of afferent signals in the Kohnstamm phenomenon comes from its similarity to the Tonic vibration reflex (TVR).

The TVR is induced by vibrating the muscle tendon at 80–100 Hz for around 30 s, causing the activation of muscle spindles (Duclos et al. 2007; Gilhodes et al. 1992; Mathis et al. 1996). This produces an involuntary contraction of the muscle, resulting in a similar kinematic and EMG profile to the Kohnstamm phenomenon (Gilhodes et al. 1992; Mathis et al. 1996), along with overlapping activations in the cortex (Duclos et al. 2007) and the elicitation of comparable descriptions of the subjective experience (Hagbarth and Nordin 1998). If the Kohnstamm phenomenon and TVR are the same phenomenon, it would follow that afferent signals from muscle spindles are the common origin (although signals from Golgi tendon organs could not be completely dismissed). However, there have been no experiments attempting to dissociate the Kohnstamm phenomenon and TVR. Establishing if this afferent signal is necessary for the Kohnstamm phenomenon, though important, does not reveal what central mechanisms in the spinal cord or brain may underlie the generation of the aftercontraction.

The Kohnstamm phenomenon may also be related to the lean aftereffect. The lean aftereffect refers to the finding that following a prolonged period (>120 s) of standing on a tilted surface (induction phase), participants will continue to lean forward (test phase) when returned to a flat surface (Walsh 1973; Gurfinkel et al. 1981). The lean aftereffect was recently shown to occur when an oscillating induction is used (platform oscillating from 4 to 10°, toes up), and to be present regardless of whether a rigid or oscillating surface is used during the test phase (Wright 2011). It was argued that a variable induction ruled out explanations of the lean aftereffect based on peripheral adaptation. Instead, the author suggested that the induction caused a central shift in a postural reference frame, which caused a change in descending motor signals (Wright 2011). Nevertheless, as with the Kohnstamm phenomenon, it is likely that afferent signalling during the induction procedure is necessary to drive the putative central adaptations.

Determining what afferent signals reach the cortex during the aftercontraction can be tested via position sense of the limb (Kuehn et al. 2015; Longo and Haggard 2010; Matthews 1933; Proske and Gandevia 2009; Stuart et al. 1970). It is known that isometric contractions and changes attributed to muscle thixotropy alter position sense (Tsay et al. 2014). However, it has also been found that sustained, isometric contractions do not reduce pointing accuracy during a voluntary movement (Heide and Molbech 1973), although they do reduce the participant’s confidence in their responses. However, it should be noted that rapid voluntary movements would have disrupted the sensory conditioning caused by the initial voluntary, isometric contraction. Of more relevance, it has been found that, while postural persistence (turning the head to the right for 10 min) produces a bias in position sense, this was not found after inducing a neck turning aftercontraction (Howard and Anstis 1974). Indeed, positional after-effects have been reported to be unrelated to the Kohnstamm phenomenon in terms of how their duration varies across individuals (Cratty and Duffy 1969). Thus, there is some evidence that afferent signals from the involuntarily contracting muscle are processed in the cortex not as purely peripheral sensory events, but as corollaries of voluntary action.

To determine what sensory signals reach the brain during the Kohnstamm phenomenon, it is especially informative to explore how sensory inputs affect the aftercontraction. Contractions from other muscles in the body can alter the aftercontraction. Concurrent voluntary dorsiflexion of the foot and weighted ipsilateral arm inductions has been seen to increase the size of hip aftercontractions (Sapirstein et al. 1937). It has also been observed that bilateral aftercontractions of the lateral deltoid were smaller than those that were unilateral (Paillard 1951). Flexion of the trunk and neck towards the involuntarily rising arm has been observed to decrease the size of the aftercontraction, while flexion away had the opposite effect (Bellincioni 1926). EMG was not recorded in any of these studies, making it impossible to know if the activity of the agonist muscle was constant across conditions. However, recent studies have found that despite matched inductions (forces and duration), sitting and lying supine are associated with significantly reduced aftercontraction of the deltoid muscle relative to standing (Ghafouri et al. 1998; Lemon et al. 2003). These findings could all be explained by efference-related changes in central regions.

Contrastingly, a few notable experiments have employed purely sensory perturbations. Building on the surprising finding that the aftercontraction sometimes transfers from one muscle to another (Craske and Craske 1985, 1986; Gurfinkel et al. 1989), it has been found that this switching can be triggered by visual input. By having participants position their arm so that both extension and flexion was possible, it was demonstrated that under diffuse light conditions (but not darkness) opening and closing the eyes led to the aftercontraction switching from the biceps to the triceps and vice versa in 10/14 participants tested (Gilhodes et al. 1992). The effect was also shown in the same participants for the TVR. EMG recordings showed that switching was not due to muscle activity during induction. Further work has confirmed visually induced switching in other muscle groups (Ghafouri et al. 1998). Integration of ascending sensory signals may occur in tonigenic sub-cortical structures such as the reticular formation (Gurfinkel et al. 1989), which is known to be strongly activated by visual input (Mori et al. 1980). However, cortical accounts cannot be ruled out. The basis of these remarkable effects is not fully understood. Such results may appear like auto-suggestion or experimenter effects. However, spontaneous muscle switching has been independently replicated (Meigal et al. 1996). Furthermore, shining strong light into participant’s eyes from the left has been shown to reduce a right arm aftercontraction (during bilateral aftercontractions), while shining light from the right reduces the left arm aftercontraction (Wells 1944).

**Fig. 3:**
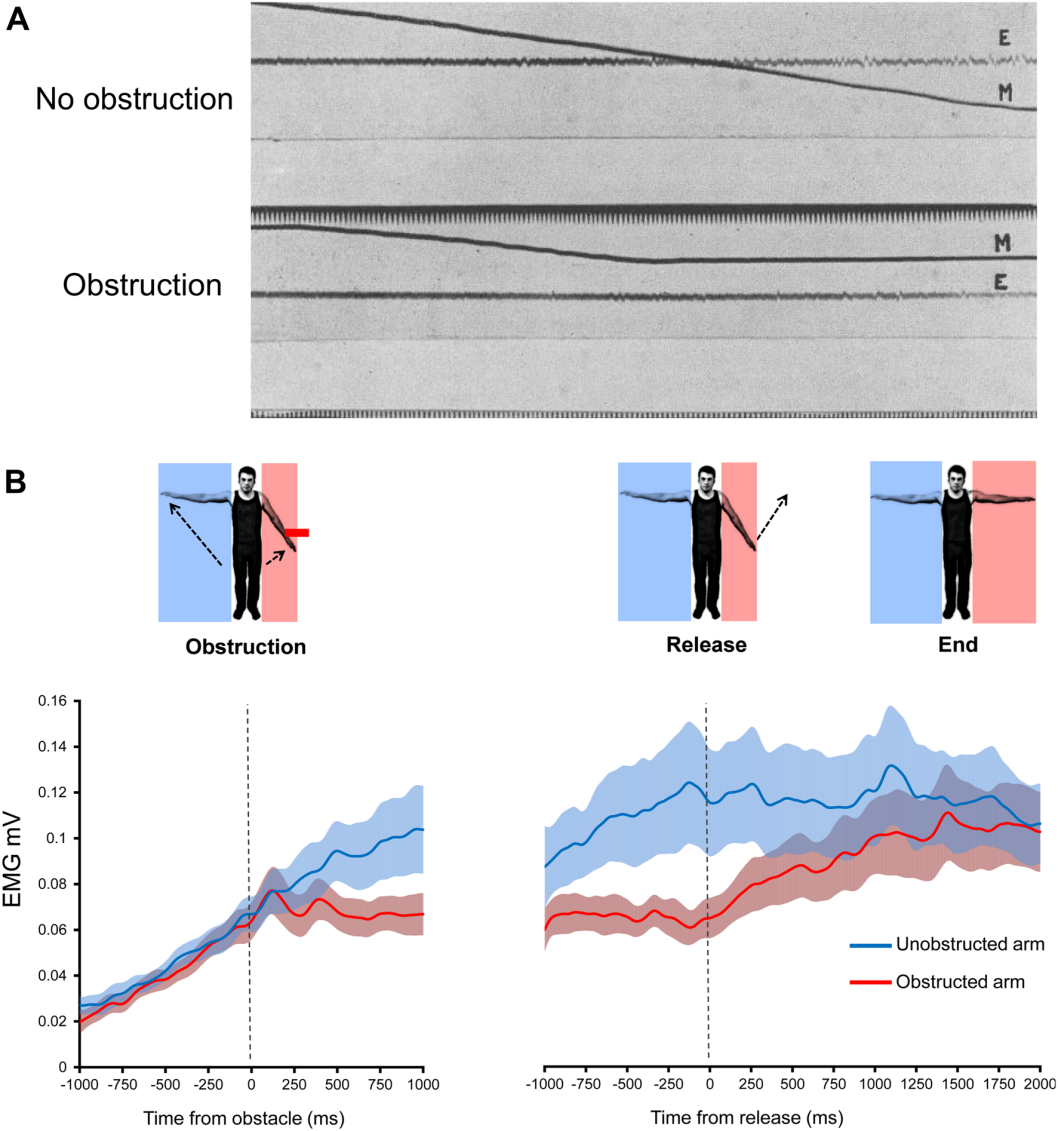
Results of physically obstructing of the aftercontraction. The first panel **a** shows an early experiment to determine whether physical obstruction of the aftercontraction resulted in a cessation of muscle activity. Arm position (*lines* labelled M) and electromyography (*lines* labelled E) are shown when no obstacle was present (*upper graph*) and when the arm was obstructed at around 20° of abduction (*lower graph*). Only single traces could be recorded at that time, but the experiment confirmed that electrical activity could be detected by a string galvanometer following obstruction, disproving an earlier claim that electrical activity detected during the aftercontraction was due to the movement itself, rather than a reflection of involuntary muscle activity (Adapted from Forbes et al. 1926) The second panel **b** shows the results of a more recent experiment involving unpredictably obstructing one arm for 2 s during a bilateral aftercontraction. Group average EMG is shown (*error bars* show SEM). It was found that physical obstruction caused a significant reduction in the slope of the aftercontraction EMG, relative to no obstruction, indicating that the output of the Kohnstamm generator is modified by afferent signals. Upon removal of the obstacle the previously obstructed arm immediately resumed its previous involuntary abduction and accompanying pattern of increasing EMG. Final arm angle and EMG level was the same as for the never obstructed arm, indicating that afferent information did not alter the state of the Kohnstamm generator itself, but rather only attenuated its output (Adapted from De Havas et al. 2015). (Color figure online)

It is not clear how afferent input from the muscle influences the aftercontraction. Proprioceptive input in the form of tendon vibration applied to the ipsilateral arm can increase the velocity of a contralateral aftercontraction (Brun et al. 2015). In addition, reducing the weight of the arm using a counterweight was found to reduce EMG during the aftercontraction (Parkinson and McDonagh 2006). This effect may be due to reduced afferent discharge from Golgi tendon organs (GTO) or lower spindle firing due to reduced arm velocity. On that view, the control of the Kohnstamm movement would involve a putative positive feedback loop linking GTO discharge to α motor neuron drive, or the established negative feedback loop linking spindle discharge to α motor neuron drive. The most direct way to determine the effects of afferent input on the Kohnstamm generator is via physical obstruction of the involuntarily rising arm. An early report involving single traces obtained by a string galvanometer (Fig. 3a) suggested that obstruction does not end the aftercontraction or reduce central innervation (Forbes et al. 1926). Furthermore, it has been shown that EMG during the aftercontraction is proportional to the angle of the rising arm (Adamson and McDonagh 2004). Here, the arm was obstructed at 15, 35, 55, and 70° of abduction. Mean EMG at contact with obstacle increased across these positions, differing significantly between 15° and 70°. Single traces also appeared to show that at the point of contact with the obstacle, the EMG remained constant. This was confirmed by a recent investigation (Fig. 3b), which found that obstructing the aftercontraction caused the increasing linear trend in agonist EMG to reach a plateau level (De Havas et al. 2015). Thus, afferent signalling from the agonist muscle can affect the aftercontraction. Removal of the obstacle caused an immediate return to the previous pattern of increasing EMG, resulting in a resumption of the involuntary movement and a final arm angle and EMG level similar to that achieved in trials without any obstruction. This suggests that the afferent signals resulting from hitting the obstacle did not alter the state of the brain circuits that generate the Kohnstamm phenomenon. Rather, it implies that the unchanging, afferent-independent output from this putative Kohnstamm generator was first integrated with incoming afferent signals, so that the EMG level reflects the combination of both influences. Analysis of single trials showed that the agonist EMG was not flat during the obstruction period, but showed an oscillatory pattern, consistent with a constant motor command accumulating, but then being repeatedly reset by an afferent signal (De Havas et al. 2015).

Thus, a variety of afferent signals interact in central regions to modify the Kohnstamm phenomenon. Position signals from the contracting muscle seem to combine with the drive from the Kohnstamm generator to set the level of the motor command, and thus the observed EMG. It is not known how strong of an effect such signals have during an unobstructed aftercontraction, and if these afferent signals form a negative-position control feedback loop with the Kohnstamm generator.

### What is changing in the brain?

A key question regarding both the mechanisms behind the Kohnstamm phenomenon and its relevance to voluntary action is the extent to which changes can be detected in the brain. Subcortical (Foix and Thevenard 1923; Rothmann 1915) and cortical (Salmon 1915, 1916, 1925; Sapirstein et al. 1936, 1937, 1938) theories have been advanced. Early cortical explanations involved a persistence of the voluntary movement. This was hypothesised to be a kinaesthetic after-image (Salmon 1916, 1925), in modern terms this is akin to a reactivation of the voluntary motor programs used during the induction in modern terms. Alternatively, the aftercontraction was hypothesised to result from a persistence of the excitatory state of the motor cortex caused by the initial strong isometric contraction (Sapirstein et al. 1937, 1938). It was observed that the aftercontraction was diminished, but present, in patients with Tabes dorsalis (Kohnstamm 1915; Rothmann 1915; Salmon 1916, 1925), a condition resulting from untreated syphilis, which caused demyelination of proprioceptive pathways. Sapirstein, Herman, and Wechsler (1938) studied 12 tabetic patients, all of whom lacked basic proprioception and showed no knee jerk response to a tendon tap. A normal aftercontraction was observed in 10 of the patients, and there was no correlation between symptom severity and aftercontraction size. The authors also examined seven patients with Parkinson’s and found that they all exhibited strong, prolonged aftercontractions, and that in some cases, tremors were visibly reduced during the movement. This extended duration was noted by earlier authors (Laignel-Lavastine et al. 1927; Salmon 1916, 1929; Selionov et al. 2013). Amongst patients with hemiplegia, they found that while that the spinal reflexes were hypersensitive on the affected side of the body, aftercontractions were markedly reduced. Others noted this reduction (Rothmann 1915; Salmon 1916, 1925). However, it could be that these patients could not produce adequate voluntary induction contractions (Salmon 1929). Finally, a single case of abnormal cerebellar development was studied and it was noted that the aftercontraction was strong, but unusually jerky in character. Together, the results suggest that Kohnstamm generation is cortical and that it is modified by sub-cortical structures in a similar fashion to voluntary movement.

Other evidence purporting to demonstrate a cortical origin is harder to interpret. Bromides (2 gm sodium bromide) were found to reduce the size of the aftercontraction, while other drugs that are known to have less effect on cortical function had no effect (Sapirstein et al. 1936). The effect of bromides was found to be ameliorated by caffeine (Sapirstein et al. 1936), which, along with alcohol has been reported to increase the aftercontraction (Danielopolu et al. 1921; Forbes et al. 1926; Matthaei 1924a). However, without adequate control experiments and EMG recordings, it is impossible to know if the drugs had a direct effect on the aftercontraction.

Similarly, there is a notable consensus amongst authors that personality traits such as positivity and emotional reactivity were correlated with large aftercontractions, while negativity and low reactivity were associated with smaller aftercontractions (Kohnstamm 1915; Laignel-Lavastine et al. 1927; Salmon 1925, 1929; Sapirstein 1948, 1960; Sapirstein et al. 1937). Indeed, Sapirstein (1948, 1960) employed the aftercontraction as a diagnostic tool within the field of psychiatry, testing hundreds of individuals, and observing that this relationship between traits and the aftercontraction persisted when they were amplified into the psychiatric range. The appearance of the aftercontraction predicted the recovery of patients, while its disappearance predicted periods of worsening mental health. Unfortunately, without physiological recordings, it is impossible to discount task compliance as the significant variable. There have been no modern experiments on the topic.

**Fig. 4:**
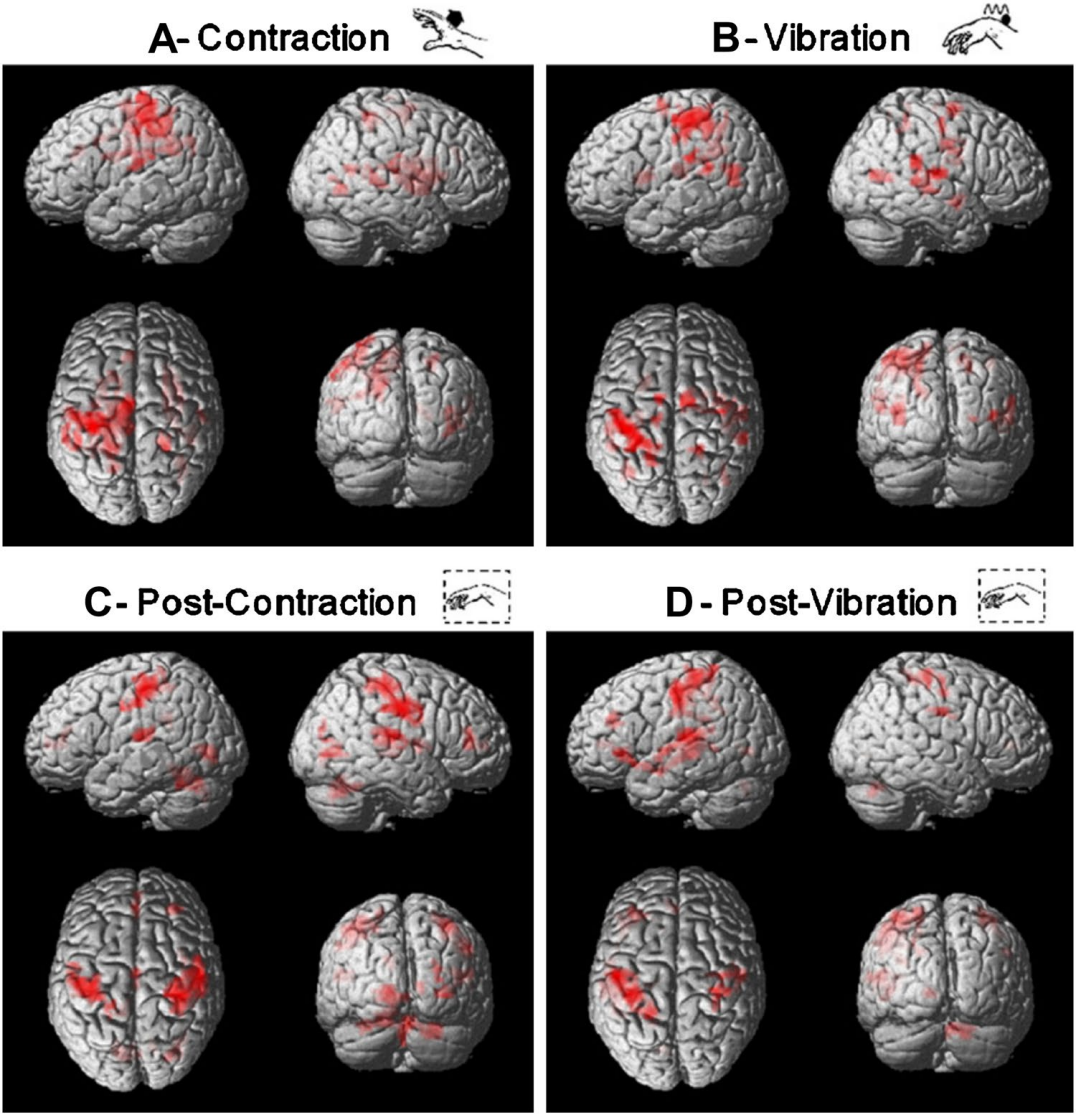
Brain regions active during Aftercontraction and TVR. Brain regions showing a significant increase in BOLD-signal in 11 subjects during **a** voluntary induction contraction of wrist extensor muscle, **b** vibration of wrist extensor tendon, **c** involuntary aftercontraction of wrist extensor muscle (here referred to as a post-contraction), and **d** post-vibration response (more commonly known as TVR) in contrast with a rest period (no movement; false discovery rate, *P* < 0.005). Note the large regions of sensorimotor cortex active during the Kohnstamm aftercontraction (Adapted from Duclos et al. 2007). (Color figure online)

Historically, direct attempts to show a cortical origin were confined to animal experiments. Sustained stimulation of the monkey motor cortex produced prolonged contractions of the muscle, but these innervations could not be distinguished from those during seizures (Sapirstein 1941). However, recent fMRI work in humans has confirmed the involvement of the cortex in the Kohnstamm phenomenon (Fig. 4). Duclos et al. (2007) had participants first experience a small wrist aftercontraction, and then a TVR, involving the extensor muscle tendon at the wrist level. In the scanner, these movements were compared to rest and voluntary movements. No significant differences were found between the aftercontraction and TVR. Both activated an extensive network of regions including primary sensory and motor cortices, premotor cortex, cingulate cortex, inferior and superior parietal cortex, insula, and the vermis of the cerebellum. In the contrasts between aftercontraction and voluntary movement, the aftercontraction was associated with greater activity in bilateral cerebellar vermis, right premotor cortex, cingulate cortex, supramarginal gyrus, and the thalamus. Voluntary movement involved significantly higher activity in the left supplementary motor area, primary sensory and motor cortices, and posterior parietal cortex and insular.

The finding that the Kohnstamm phenomenon is associated with activity throughout the cortex has been replicated (Parkinson et al. 2009). Both studies found that the anterior cingulate cortex showed prominent activity during the aftercontraction. This could be due to the region’s well-documented role in error monitoring (Carter et al. 1998; Taylor et al. 2007) or a more direct involvement in generating movement commands (Ball et al. 1999; Paus 2001), perhaps via the modulation of postural centres in the brainstem (Takakusaki et al. 2004). Both studies found high levels of activity in the parietal lobes, cerebellum, primary motor cortex and premotor regions (Duclos et al. 2007; Parkinson et al. 2009). The supplementary motor area, which is a key structure in goal-directed movement programming (Geyer et al. 2000; Tanji 1996), was either only active during voluntary movement (Duclos et al. 2007), or active to the same degree across aftercontraction and voluntary movement (Parkinson et al. 2009). The cortex is clearly involved in the Kohnstamm phenomenon. However, activity in the cortex could be epiphenomenal, rather than a direct reflection of the Kohnstamm generator itself. For example, it could reflect sensory feedback from the moving limb, or even mental imagery triggered by the unusual experience (Decety 1996).

**Fig. 5:**
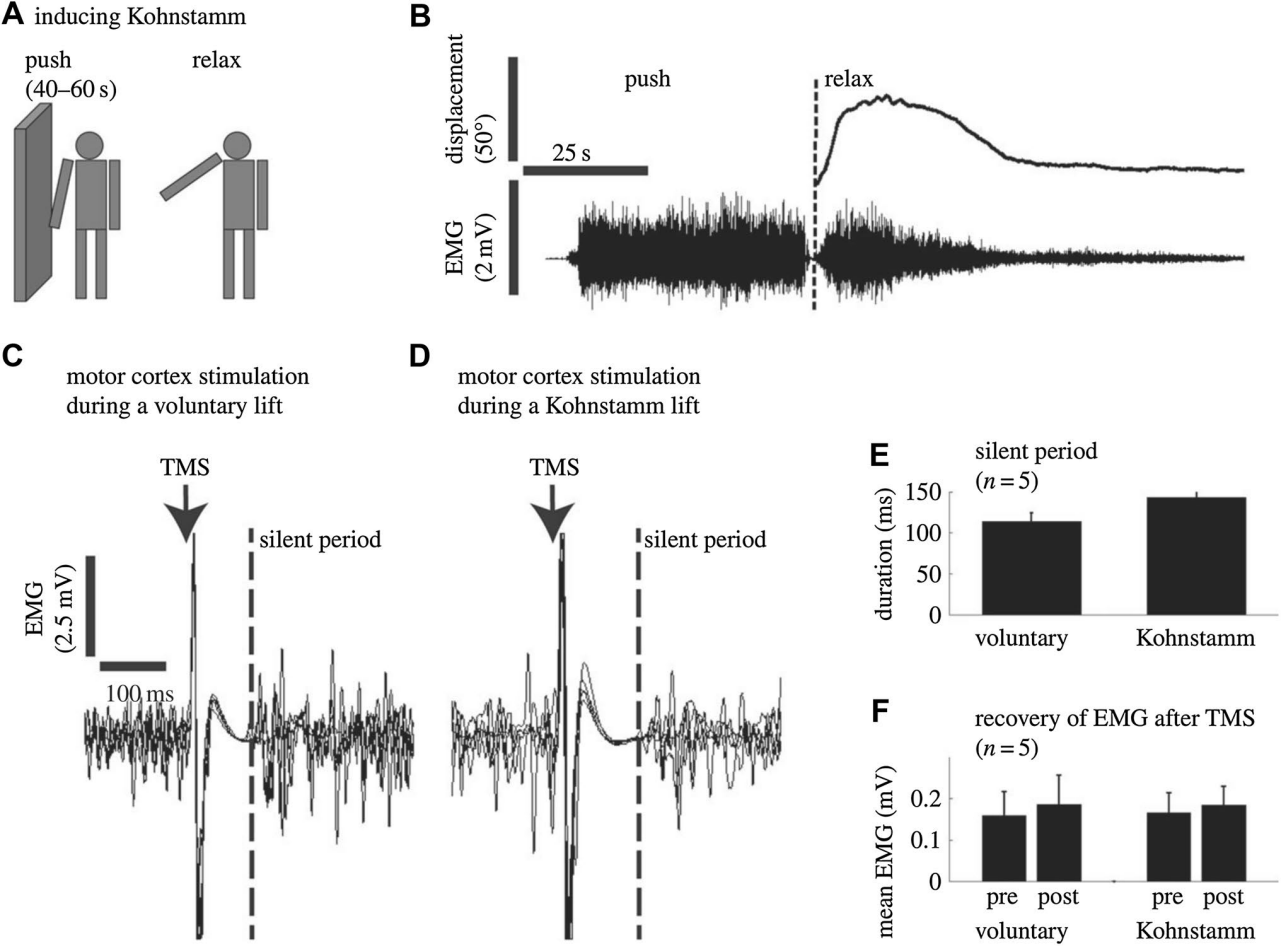
Applying TMS to M1 during aftercontraction shows cortical involvement in Kohnstamm phenomenon. A Kohnstamm aftercontraction was induced by having the participants push against a wall and then step away and relax the deltoid muscle (**a**). Kinematic and EMG traces of the Kohnstamm induction and aftercontraction are shown from a single representative participant (**b**). TMS of the motor cortex during aftercontraction (**d**) and matched voluntary movements (**c**) results in a prolonged silent period, suggesting a cortical origin (representative participant’s data). Mean muscle silent period duration following application of TMS did not differ across aftercontraction and voluntary movement conditions (**e**). Muscular contractions made a full recovery after the silent period for both Kohnstamm aftercontractions and voluntary movements (**f**). Adapted from Ghosh et al. (2014)

More direct evidence comes from the effects of attention, mental imagery and visual input. Inductions involving isometric contractions of the elbow and shoulder can produce aftercontractions in the ipsilateral hip and knee (Craske and Craske 1985). The effect also worked in the other direction and involved having participants name the non-induction limb repeatedly and silently at the point of relaxation. It was confirmed that this effect of attention could induce transfer of aftercontraction from one arm to the other (Craske and Craske 1986). Intriguingly, it was also found that imagining pushing outwards for 60 s could also result in an aftercontraction of the shoulder. The above experiments did not involve verification of transfer by EMG and featured a reasonable degree of unexplained spontaneous arm movements, indicative of an expectation effect. However, the previously cited experiments showing that visual input can induce muscle switching (Ghafouri et al. 1998; Gilhodes et al. 1992) do not suffer from this limitation. These experiments indicate that, regardless of the origin of the aftercontraction, output to the muscle must first pass through the cortex. This has been confirmed (Fig. 5). Applying transcortical magnetic stimulation to the primary motor cortex during the aftercontraction induces a silent period in the contracting agonist muscle (Ghosh et al. 2014). The silent period did not differ in terms of latency or duration from that obtained during a matched voluntary movement. Silent periods were >100 ms, which is an established indicator of cortical inhibition (Chen et al. 1999; Fuhr et al. 1991; Terao and Ugawa 2002).

Obstruction, or voluntary inhibition, of one arm during bilateral aftercontractions did not affect agonist EMG in the other arm. This suggests that each cerebral hemisphere has an independent Kohnstamm generator (De Havas et al. 2015, 2016). However, it appears that while both arms are moving there can be some signal integration between putative Kohnstamm generators in each hemisphere (Brun et al. 2015; Brun and Guerraz 2015). In sum, there is now good evidence that the aftercontraction is driven by output from the primary motor cortex. However, many questions remain regarding cortical involvement in the Kohnstamm phenomenon, with comparisons voluntary movement being particularly informative.

### What is the relationship between this involuntary movement and voluntary control?

Kinematically, the aftercontraction is identical to a slow voluntary movement. Similarly, the EMG signal is comparable to a voluntary movement of similar size and speed (Fessard and Tournay 1949; Forbes et al. 1926; Schwartz 1924; Schwartz and Meyer 1921). There is also evidence that the entire motor system shows the same level of excitability during both forms of movement. Mathis et al. (1996) applied 8–10 Transcranial Magnetic Stimulation (TMS) pulses (ISI = 8 s) to the left motor cortex during right deltoid aftercontractions and matched voluntary movements in seven healthy participants. They found that, despite the maximum abduction being lower in the aftercontraction compared to the voluntary movement (22° versus 27°), the EMG did not significantly differ (57 versus 45 mV). Importantly, there was no significant difference in the mean amplitude of Motor Evoked Potentials (MEP) elicited by the TMS (aftercontraction = 1.3, Voluntary = 1 mV). In both conditions, MEP size correlated with background EMG level, and there was no difference in the gain, latency, or dynamics of the MEPs across conditions. Interestingly, an additional benefit of rising EMG (i.e., abduction, muscle shortening) compared to falling EMG was found in 20% of voluntary trials and 30% of aftercontraction trials. These findings are complemented by the already cited imaging work which found no significant difference in the activity in the primary motor cortex during aftercontraction and matched voluntary movements (Duclos et al. 2007; Parkinson et al. 2009).

**Fig. 6:**
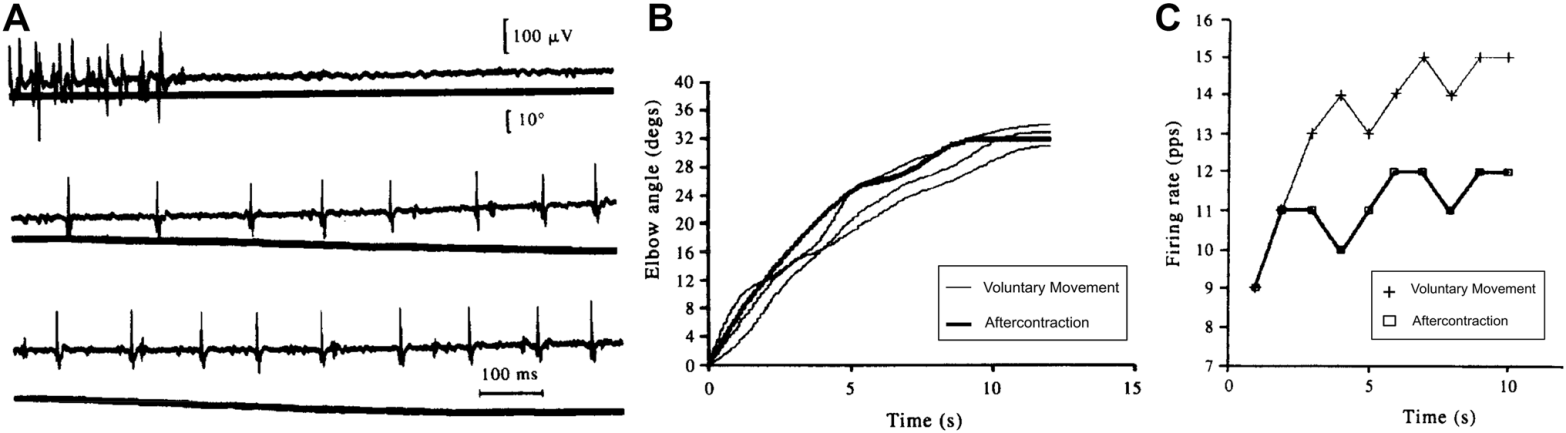
Mean firing rate of motor units significantly lower during aftercontraction compared to voluntary movements. The first panel **a** shows a raw EMG recorded in human triceps muscle showing recruitment of a motor unit during the first 2 s of an aftercontraction. *Solid line* shows elbow joint angle change. Motor unit firing rate progressively increases after the latent period, followed by a relatively steady state of firing. Aftercontractions were compared to voluntary movements of matched velocity (**b**). It was found that across participants motor units showed lower firing rates (**c**) during aftercontraction compared to voluntary movements (Adapted from Kozhina et al. 1996)

However, work using intramuscular needle electrodes does not fully support this account. Kozhina et al. (1996) recorded single motor unit activity from the deltoid and triceps muscle in four participants during aftercontraction and matched voluntary movements (Fig. 6). The standard latent period of muscle silence was seen after the Kohnstamm induction (triceps = 1.4, deltoid = 1.5 s), followed by a 1–2 s when the firing rate increased, before remaining constant for the rest of the aftercontraction. Standard deviation of spike rate did not differ across voluntary movements and aftercontraction. In addition, EMG recordings from the antagonist muscle (bicep) during tricep contractions did not differ. However, the mean firing rate of motor units was significantly lower during aftercontraction (12 pps) compared to voluntary movements (14 pps), despite the velocity and amplitude of the voluntary movements never exceeding that seen during aftercontraction. Thus, while the motor cortex and descending pathways do not differ in terms of gross excitability across aftercontraction and matched voluntary movements (Mathis et al. 1996), this does not preclude subtle differences in the state of motoneurons. It may be that the aftercontraction involves adaptations in motoneurons, which allow the same movement to be achieved with a lower firing rate (Kozhina et al. 1996).

Central to understanding involuntary and voluntary motor control is determining how the two forms of movement interact. The Kohnstamm phenomenon may feel subjectively like it is uncontrollable, yet the arm can be easily brought under voluntary control by the participant (Kohnstamm 1915). Small voluntary movements in the direction of the aftercontraction may actually aid the appearance of the phenomenon (Salmon 1916), although the precise timing of this effect has not been investigated. The aftercontraction does not prevent simultaneous voluntary movements of the same muscle (Fessard and Tournay 1949; Hick 1953; Shea et al. 1991), with voluntary movements apparently superimposed over the involuntary one (Hick 1953). Furthermore, hip aftercontractions have been shown to dramatically alter the attempts of blindfolded participants to walk in a straight line (Ivanenko et al. 2006). The effect was always in the direction of the aftercontraction and disappeared when participants stepped in place on a treadmill, suggesting specificity in the movement programs affected. However, the above experiments have limited interpretability, since the observed behaviour does not separate the involuntary and voluntary contributions to the movement. Other voluntary movements have been found to have an inhibitory effect on the aftercontraction. Rapid voluntary movements during the latent period can prevent the aftercontraction from emerging (Duclos et al. 2004; Hutton et al. 1987). Paillard (1951) noted that sudden voluntary upwards movements of one arm cause transient inhibition of an aftercontraction occurring in the other arm. These effects may be due to a form of ‘resetting’ of the sensorimotor system caused by the voluntary movement or a form of top-down motor inhibition of the developing aftercontraction. Alternatively, the contralateral movement may just superimpose a postural adjustment on the other arm in addition to the aftercontraction.

The possibility of voluntarily stopping the aftercontraction has always been known about (Kohnstamm 1915). Early reports indicated that it was easily possible to stop the aftercontraction during the latent period (Forbes et al. 1926; Pinkhof 1922). Indeed, inhibition of one arm during latent period apparently does not affect the aftercontraction in the other arm (Paillard 1951). Voluntarily stopping the arm and holding it stationary during the involuntary movement was reported to be more difficult (Forbes et al. 1926). Actively adducting the arm against an abducting aftercontraction does not appear to extinguish the phenomenon (Fessard and Tournay 1949), with the effect that the arm sometimes begins to rise again once it has been brought back to the start position. These findings suggest an intriguing possibility: that voluntary inhibitory commands can modify involuntary movements.

**Fig. 7:**
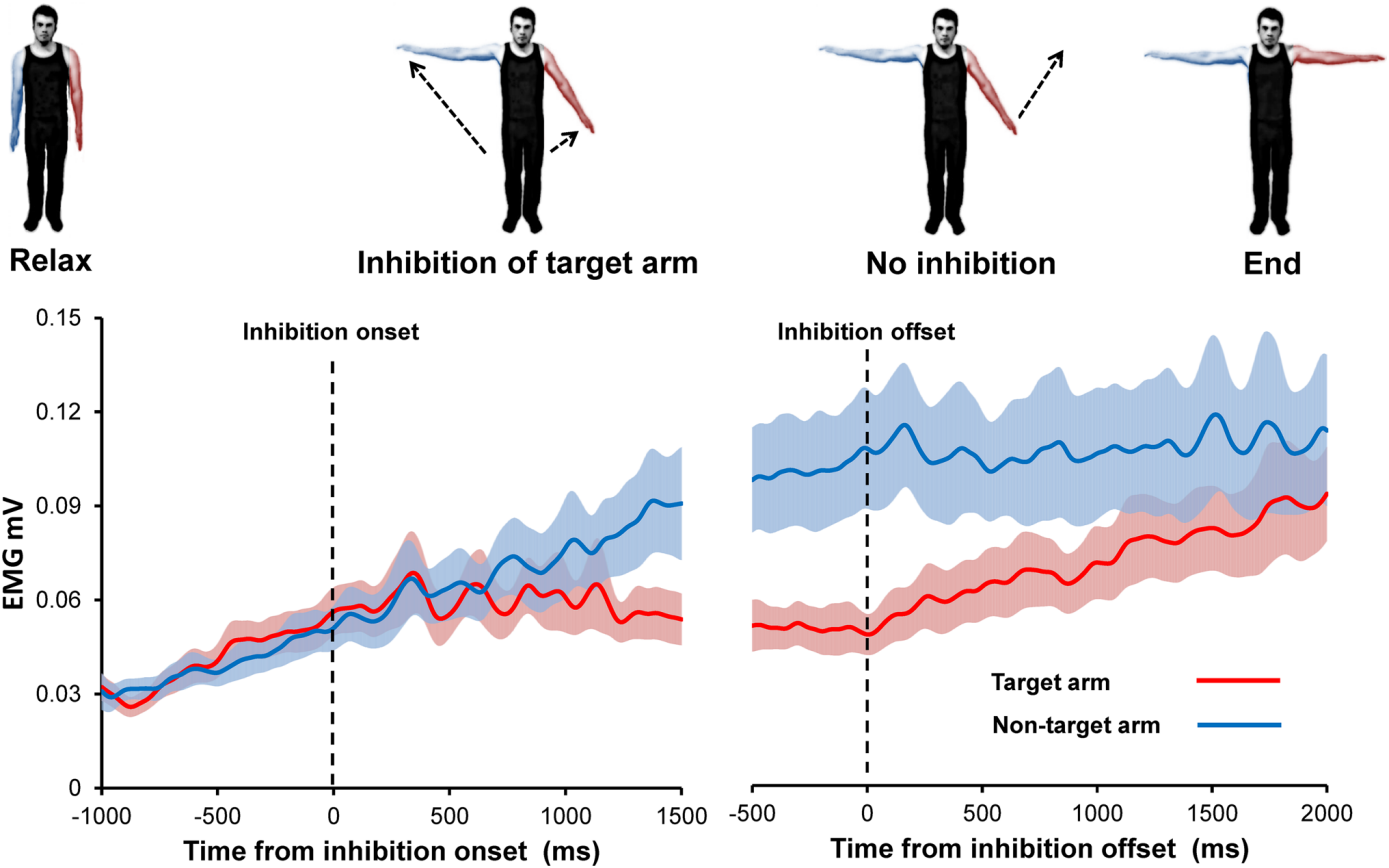
Voluntary inhibition of Kohnstamm aftercontraction. The effect of inhibiting, and releasing inhibition, of a single ‘target’ arm during bilateral Kohnstamm aftercontraction on rectified, smoothed deltoid EMG. Dashed lines show time of inhibition onset and offset. Error bars show SEM. Note the significant increase in EMG for the non-target arm relative to the plateauing of EMG in the target arm, beginning approximately 500 ms after the instruction to inhibit. After participants were instructed to stop inhibiting, target arm EMG increased and the arm began to involuntarily rise once more. Final arm angle and EMG level was the same for both arms across participants, indicating that the Kohnstamm generator itself was not modified by voluntary inhibition (Adapted from De Havas et al. 2016). (Color figure online)

Ghosh et al. (2014) verified these observations. Following an aftercontraction of the lateral deltoid, participants were randomly instructed ‘gently bring the arm back down and actively keep it down’. They did this without the use of the antagonist muscle (pectoralis). After ‘holding’ the arm down for 1–3 s, it spontaneously rose, albeit with reduced EMG relative to the first aftercontraction. This suggests something akin to a ‘negative motor command’ can be sent to oppose the upward drive from the Kohnstamm generator. Such commands may originate from ‘negative motor areas’ upstream of the primary motor cortex. Several cortical areas have been reported to cause slowing and cessation of movement when directly stimulated (Filevich et al. 2012; Brown and Sherrington 1912). This putative negative motor command appears not to permanently override the Kohnstamm generator (Fig. 7). After a brief (~ 2 s) period of inhibition (where the participant was instructed to keep the arm stationary mid-way through an aftercontraction), the arm begins to immediately rise once the instruction to inhibit is removed, and reaches the same final angle as if it had not been inhibited (De Havas et al. 2016). If the inhibitory command directly affected the Kohnstamm generator one would expect a delay in the resumption of movement and a reduction in the final arm angle. Instead, it seems the putative negative motor command is integrated with the excitatory output from the Kohnstamm generator at a lower level, perhaps M1 (De Havas et al. 2016). Further work is needed to determine precisely how the Kohnstamm phenomenon relates to voluntary movement at the level of control principles and physiology.

### Control principles underlying the Kohnstamm phenomenon

The control principles underlying the Kohnstamm phenomenon have been investigated by systematically varying the induction contraction. Duration (Fessard and Tournay 1949; Matthaei 1924a) and amplitude (Allen 1937; Allen and O’Donoghue 1927; Holway et al. 1937; Matthaei 1924a) of the induction contraction are positively correlated with the amplitude of the aftercontraction in terms of the angular displacement of the limb. This holds for durations up to ~2 min, when the aftercontraction begins to decrease due to fatigue (Salmon 1929). Attempts were made to characterise this relationship in terms of a log function (Allen and O’Donoghue 1927) and power function (Holway et al. 1937). However, these efforts were based on inadequate samples and were confounded by the fact that repeating many Kohnstamms within a short space of time may initially produce reinforcement, resulting in increased aftercontraction size (Sapirstein et al. 1937) and then fatigue, resulting in decreased aftercontraction size (Danielopolu et al. 1921; Sapirstein et al. 1937; Zigler et al. 1948). Other authors have observed possible augmentation effects resulting from performing multiple Kohnstamms, interspersed with 20 min rests (Allen and O’Donoghue 1927), rendering the possibility of obtaining simple laws for aftercontraction size unlikely. A more recent attempt, using a larger sample size and modern recording equipment, found that once the duration of the induction reaches a certain threshold (~45 s), the size of the aftercontraction is related to the size of the muscular contraction (Brice and McDonagh 2001), with for example, 60 s of 30% deltoid MVC producing 50° of angular displacement of the arm, and 70% producing 92° on average.

#### Persistence of motor activity

The above evidence can be explained by the Kohnstamm generator being a persistence of the voluntary command (Salmon 1925; Sapirstein et al. 1937). This theory (see Table 2) is consistent with reports of aftercontractions in patients with deafferentation due to Tabes dorsalis, but reduced aftercontractions in patients with hemiplegia (Kohnstamm 1915; Rothmann 1915; Salmon 1925; Sapirstein et al. 1938). Indeed, it also seems consistent with reports that muscle length during induction does not seem important (Forbes et al. 1926; Hagbarth and Nordin 1998). On such an account, any modulation in the structure of the inducing contraction would be expected to be present in the aftercontraction. Previous literature on varying the induction gives little indication of the control principles of the Kohnstamm generator. There have been no studies where the induction contraction is systematically varied, while controlling for the total amount of muscle activity.

A number of findings disagree with ballistic, feedforward control. First, it is difficult to reconcile the latent period of several seconds with a simple replaying of the motor command (Csiky 1915; Kozhina et al. 1996; Salmon 1929). If the Kohnstamm represents perseveration of a voluntary motor command, why is there a delay before perseveration starts? Early suggestions, that the latent period is actually the time taken to release an unspecified inhibitory control (Kohnstamm 1915), are not supported by the subjective sensation of simply relaxing. This contrasts with the sensation of active inhibition when participants voluntarily stop the aftercontraction (De Havas et al. 2016; Forbes et al. 1926; Ghosh et al. 2014). Furthermore, theories of persistence of excitation within the motor cortex (Sapirstein et al. 1937) are not supported by the finding that the size of cortical evoked potentials is small and proportional to EMG during the Kohnstamm latent period (Mathis et al. 1996). More recently, it has been shown that afferent feedback from hitting an obstacle has a strong effect on agonist EMG during aftercontractions (De Havas et al. 2015), indicating that the Kohnstamm phenomenon does not involve ballistic, feedforward control. It is also hard to reconcile simple persistence of motor excitation accounts with the finding that unidirectional leg and arm inductions can produce complex patterns of rhythmic leg and arm movement (Selionov et al. 2013, 2009; Solopova et al. 2016), and with the finding that visual input can cause muscle switching (Ghafouri et al. 1998; Gilhodes et al. 1992). Nevertheless, it remains an open question to what extent the Kohnstamm generator replays in feedforward fashion the same motor commands used to generate the voluntary contraction that induces the Kohnstamm.

#### Negative position feedback

Once the aftercontraction contraction has begun, muscle activity could be controlled via negative position feedback from muscle afferents (Table 2). It is known that there exists a tight coupling between the arm angle during the aftercontraction and EMG (Adamson and McDonagh 2004; De Havas et al. 2015). Indeed, such positional theories are consistent with a peripheral origin of the Kohnstamm phenomenon, whereby the induction phase would lead to some change in a peripheral signal that drives motor circuits. One model views the Kohnstamm phenomenon as a form of proportional-integral-derivative (PID) control, similar to equilibrium point control (Feldman 1986; Bizzi et al. 1992), proposed for both stretch reflexes and voluntary actions. For such control, a central motor signal setting the equilibrium point of the muscle would result in a follow-up servo contraction of the muscle, causing a movement towards that position. Alternatively, the equilibrium point might move gradually over time, defining a virtual trajectory (Bizzi et al. 1984; Hogan 1985). Here, increased aftercontraction from longer and more powerful induction contractions would be explained by greater peripheral adaptation. A virtual trajectory account seems broadly consistent with the existing electrophysiological evidence of increasing muscular activity with movement (Adamson and McDonagh 2004; Fessard and Tournay 1949; Kozhina et al. 1996). Involvement of the motor cortex (Duclos et al. 2007; Ghosh et al. 2014; Parkinson et al. 2009) would be interpreted as being a proportional response to the ‘abnormal’ afferent inflow, existing within normal transcortical control loops. Here, silence in the muscle during the latent period (Kozhina et al. 1996) must be the time required for a sufficiently uniform afferent volley to reach the cortex, so that an efferent response is triggered.

An obvious way to test the position control theories of the Kohnstamm phenomenon is to determine how physical obstruction of the aftercontraction affects motor output. Position control theories predict that EMG should persist despite physical obstruction, and that involuntary arm movement should reach a fixed final position once the obstacle is removed. Existing experiments using this technique suggest that obstruction does not abolish the aftercontraction (Adamson and McDonagh 2004; Forbes et al. 1926). However, neither experiment examined the time course of the EMG across participants in response to the obstruction. Thus, these studies cannot provide strong tests of position control models of the Kohnstamm phenomenon. A more recent study (De Havas et al. 2015) did measure EMG responses. The EMG patterns observed clearly ruled out the ‘virtual trajectory’ hypothesis, according to which the equilibrium point moves gradually towards the final position. That hypothesis predicts continuous increase in EMG after onset of the obstruction, and restart of movement following release, with a force and acceleration proportional to the duration of the obstruction. Neither pattern was observed. Instead, the EMG level at the start of the obstruction was maintained throughout the duration of obstruction. Thus, this particular version of position control could be conclusively ruled out. However, across two studies it was found that briefly (~2 s) arresting the arm, either via a physical obstacle (De Havas et al. 2015) or via voluntary inhibition (De Havas et al. 2016), did not affect the final arm position of the aftercontracting arm. This final position constancy is a characteristic feature of position control schemes. Indeed, it may be that these findings only pertain to conditions where the involuntarily rising arm is fully arrested. It could be that negative position control normally operates during the aftercontraction, but that the strong afferent signal associated with an obstacle causes a switch in the control mechanism determining the level of muscle activity. However, to date only one experiment has examined perturbation of the aftercontraction without obstruction (see below), and though the results were compatible with negative position control, they were interpreted within a context of force feedback control. Further perturbation experiments are required to determine if negative position control is associated with the Kohnstamm phenomenon.

#### Positive force feedback

**Fig. 8:**
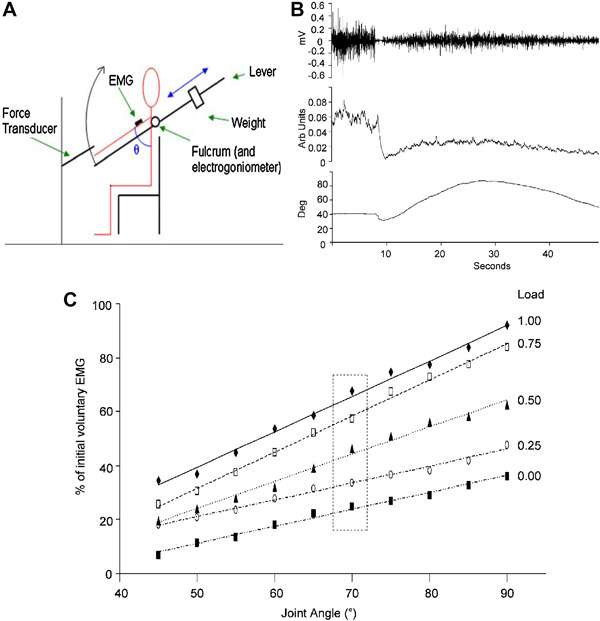
Reduced aftercontraction EMG in response to decreased muscle loading. Participants pushed upwards against the force transducer (60% MVC, 60 s) to induce an aftercontraction of the anterior deltoid muscle (**a**). A movable counter-weight attached to the arm via a lever allowed the loading on the muscle to be systematically reduced across conditions. EMG and arm angle results of a single participant are shown (**b**), including the last 10 s of the induction and the entire aftercontraction. Group average results of reducing the muscle load on EMG across joint angles are shown (**c**). A load of 1 means that the arm was of normal weight, while a load of 0 meant that the counterweight perfectly balanced the arm weight, meaning that there should have been negligible loading on anterior deltoid. Reducing the load from 1 down to 0 produced a reliable decrease in aftercontraction EMG across joint angles (Adapted from Parkinson and McDonagh 2006)

Force feedback could underlie the Kohnstamm phenomenon (Table 2). Based on work showing that EMG was lower during supine than during standing aftercontractions it was hypothesised that positive force feedback could be a critical control principle (Lemon et al. 2003). Parkinson and McDonagh (2006) tested this principle by manipulating the weight of nine participant’s arms during a shoulder Kohnstamm in the frontal plane. Arm weight was systematically reduced (100, 75, 50, 25, 0%) via the use of a moveable counter-weight on a lever attached to the arm (Fig. 8). Across conditions, participants induced the aftercontraction by pushing upwards with a force of 60% of their maximum for 1 min. It was found that mean aftercontraction EMG (as a percentage of voluntary induction EMG) was reduced across every arm angle as the weight of the arm was reduced. At a given arm angle (70°), EMG was significantly higher in the 100% arm weight (normal arm weight) condition than in the 50, 25 and 0% arm weight conditions. This was interpreted as evidence of positive feedback. As GTO signal increased throughout the abduction (due to increased muscle torque), motor efference also increased via a putative peripheral-central feedback loop. However, the design and analysis of the experiment limit interpretations. First, the counter-weight was attached throughout the induction, latent period and aftercontraction. Afferent signals during the first two stages could establish central adaptations, which underlie the EMG reductions observed. Second, it is perhaps problematic that all EMG values during the aftercontraction were referenced to the mean EMG during induction rather than an independent maximum contraction. This analysis may have been performed to control for the fact that trial order was not randomised across conditions. However, the assumption of a linear relationship between induction size and aftercontraction has numerous caveats (Brice and McDonagh 2001; Salmon 1925). It would have been preferable to first verify that the inductions did not differ across conditions and then look for changes in the aftercontraction EMG as a percentage of MVC. Velocity of arm movements was not reported, so no inferences can be made about shoulder torque or spindle firing rate across conditions. Even if velocity was matched across conditions, decreased muscle loading might produce lower spindle firing rates due to alpha-gamma co-activation (Taylor et al. 2007; Vallbo 1970). As such, a negative position feedback model could also account for the data. Finally, the positive force feedback model is inconsistent with the finding that removal of a physical obstacle during an aftercontraction was found to produce an immediate increase in EMG (De Havas et al. 2015). Removal of an obstacle is associated with a reduction of load on the muscle, which, according to the positive force feedback model, should have lead to a decrease in EMG, rather than the observed increase.

The Kohnstamm phenomenon may represent an adaptation within tonigenic structures, which have some overlap with central pattern generators involved in repetitive actions such as walking (Craske and Craske 1986; Selionov et al. 2013, 2009; Solopova et al. 2016; Waters and Morris 1972). Complex interactions occur between muscle groups (Bellincioni 1926; Craske and Craske 1985), while sensory input can interact with the aftercontraction in surprising and divergent ways (Brun et al. 2015; Brun and Guerraz 2015; De Havas et al. 2015; Forbes et al. 1926; Ghafouri et al. 1998). As such, it may be necessary to consider hybrid models, combining both central and peripheral mechanisms, to explain the Kohnstamm phenomenon. Here, a combination of central and/or peripheral sensory processing may establish a central adaptation, which in turn interacts with subsequent sensory input during the aftercontraction. Similar models have been suggested to account for other postural phenomena such as the lean aftereffect (Wright 2011). However, it is important to first exclude central-only, or peripheral-only accounts, since they are simpler than hybrid accounts.

### Subjective experience of involuntary movement

Perhaps the most striking, yet least studied, feature of the Kohnstamm phenomenon is that while the movement looks the same as a slow voluntary contraction, it feels very different for the person to whom it is actually happening (Fessard and Tournay 1949). Participants often report feeling surprised when their limb begins to move (Craske and Craske 1985; Forbes et al. 1926), and state that the limb is floating (Craske and Craske 1985; Salmon 1915), either of its own accord (Craske and Craske 1985) or via some ‘hidden force’ (Kohnstamm 1915). Another, often vivid sensation is that the limb feels much lighter than normal (Craske and Craske 1985; Cratty and Duffy 1969; Gurfinkel et al. 1989; Hagbarth and Nordin 1998; Hazelhoff and Wiersma 1922; Kohnstamm 1915). Indeed, it has been argued that the subjective feeling of lightness is the best way to gauge the duration of the aftercontraction (Cratty and Duffy 1969). In the latter study, participants continuously reported whether their arm felt lighter or heavier than normal, reporting that the arm felt lighter for an average of 14 s. However, most subjective findings in the literature are the author’s ad-hoc recollections of participant’s self-reported phenomenology or spontaneous commentary, with few attempts to fully catalogue participant’s experiences in an unbiased manner. Conversely, substantial research has been conducted on the effects of muscle contraction history on voluntary movement force generation (Hutton et al. 1984, 1987; Knight et al. 2008; Shea et al. 1991). These findings show that prior strong contractions cause participants to overshoot target force levels.

There have been some attempts to quantify the feeling of lightness during a purely involuntary movement. Matthaei, (1924a) instructed participants to maintain an equal upward force on two springs. After inducing an aftercontraction on one arm it was found that the length of the spring held by this arm was much longer than the spring held by the non-aftercontraction arm. The size of this error was found to be proportional to the strength of the aftercontraction, rather than the amount of voluntary force used by the other arm. This experiment showed that the sense of lightness experienced during the aftercontraction is not a form of post-hoc comparison to everyday voluntary movements. However, the interpretation is limited, since no statistical results were presented. Recent work on the perception of force during the Kohnstamm phenomenon is consistent with the earlier reports. Participants reported that hitting an obstacle was associated with a greater subjective force than during matched voluntary movements (De Havas et al. 2015). In a separate experiment, the forces generated when participants replicated Kohnstamm forces with a voluntary movement were greater than when they replicated voluntary forces with a voluntary movement (De Havas et al. 2015). Again, the force of the aftercontraction was overestimated. Force perception during voluntary movement may result from the cancellation of sensory inflow against an efference copy of the movement (Blakemore and Frith 2003; Shergill et al. 2003). Perceptual overestimation of Kohnstamm forces can be explained within this model. For example, if the Kohnstamm generator does not produce efference copies, there would be nothing to cancel the sensory inflow against, and a resulting overestimation of force relative to matched voluntary movements.

**Fig. 9:**
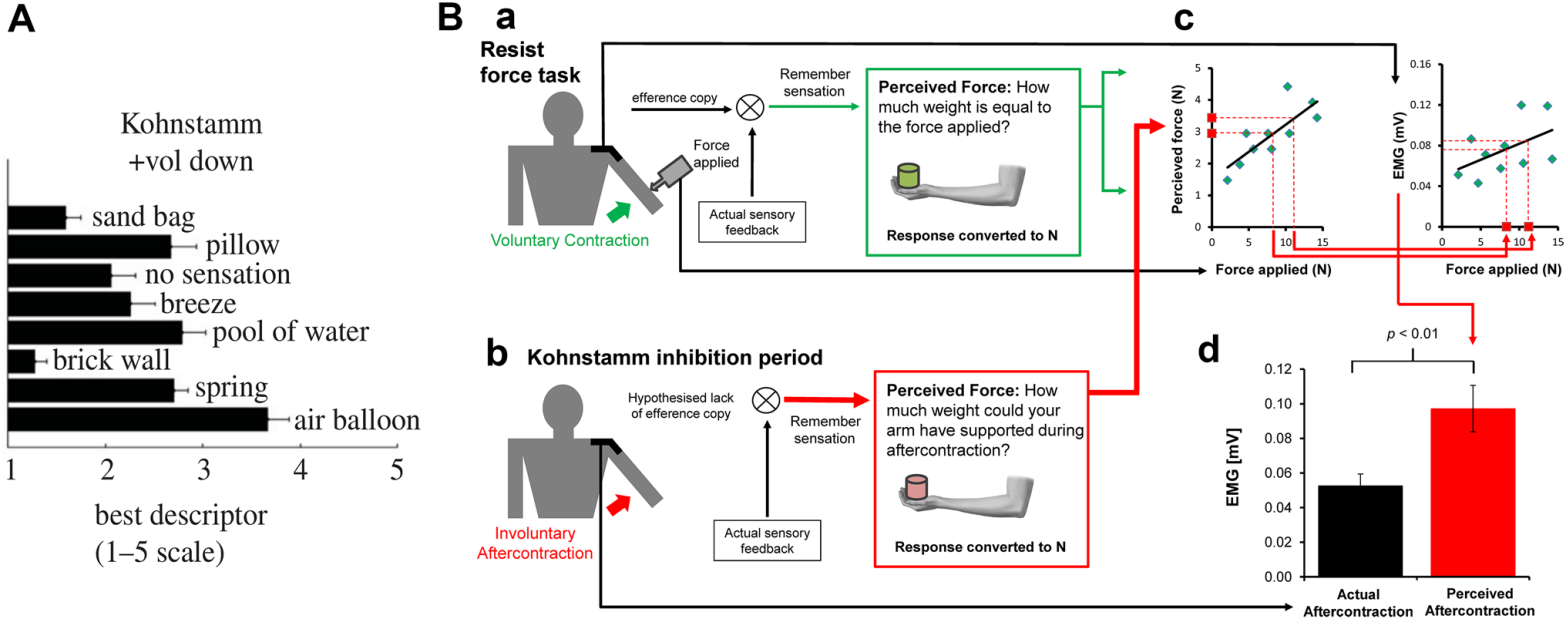
Subjective experience of inhibiting the Kohnstamm aftercontraction. The first panel **a** shows the results of an experiment in which the subjective experience of voluntarily bringing the arm down (adduction) during an aftercontraction was rated (*1* strong disagreement, *5* strong agreement). Participants clearly perceived an upward resistance, most closely resembling an air balloon (Adapted from Ghosh et al. 2014). The second panel *b* shows the results of an experiment when the subjective upward drive from the Kohnstamm generator was compared to the actual muscle contraction strength during voluntary inhibition of an aftercontraction (**b**), compared to a range of isometric voluntary contractions (*a*). Participants rated how much force their arm could support during inhibition of aftercontraction (arm held stationary, partially abducted). This rating was plotted (*c left* graph; *red squares*; single illustrative participant) together with the relation between perceived and actual force from voluntary trials (*c left* graph; *green diamonds*). Interpolating this relation allowed an estimation of the equivalent Kohnstamm forces that would be required to generate percepts similar to those on voluntary trials. The level of voluntary EMG required to generate the equivalent Kohnstamm force was calculated, using the relation between EMG and actual force for voluntary trials (*c right* graph). This perceived aftercontraction was compared to the actual level of aftercontraction EMG during the period of inhibition across participants (*d*). Subjective aftercontraction strength was significantly overestimated, suggesting the Kohnstamm generator does not produce efference copies to cancel against the sensory inflow (Adapted from De Havas et al. 2016). (Color figure online)

Another approach to studying the subjective experience of the Kohnstamm phenomenon is to ask participants about their experiences of counteracting the aftercontraction with inhibition. Ghosh et al. (2014) examined the subjective experience of participants as they lowered their arms during an aftercontraction, and compared this to the feeling of lowering the arm without an aftercontraction. In the latter condition the arm was first held in the abducted position at shoulder level for 1 min. The authors also tested the same effect in five participants who did not experience an aftercontraction after the Kohnstamm induction. Here the arm was first passively raised before being lowered voluntarily, allowing a test of the hypothesis that any subjective effects were simply a by-product of the isometric contraction. Across each condition, participants rated the sense of resistance on a scale from 0 to 50. It was found that the strongest sense of resistance was felt during the downward movement with aftercontraction. In participants with no visible aftercontraction, the ratings did not differ between the conditions. The sensation of resistance was reported to be like that of compressing an air balloon (Fig. 9a). Thus, the sensation of resistance arose as a result of the interaction between the Kohnstamm generator and normal sensory inflow from the moving limb. One explanation is that the upward lift from the Kohnstamm generator was not perceived as self-generated. If the Kohnstamm generator does not produce efference copies of the movement command, than there would be nothing to cancel against the sensory inflow, resulting in a miss-attribution of a resistance to overcome (Blakemore and Frith 2003). It is possible that that the aftercontraction rendered the downward movement less fluent, and that participants were reporting a feeling of movement jerkiness and dysfluency rather than true resistance. However, similar results have been obtained when the arm is stationary (De Havas et al. 2016). Here participants were asked to estimate how much weight their arm could support during inhibition of the aftercontraction. This was compared to a series of linearly increasing voluntary contractions (Fig. 9b). It was found that to produce the same subjective perception of aftercontraction strength required a voluntary contraction of almost twice the strength of the aftercontraction. Thus, again, Kohnstamm forces were relatively overestimated. This is consistent with the Kohnstamm generator not producing efference copies.

## A model of the Kohnstamm phenomenon

**Fig. 10:**
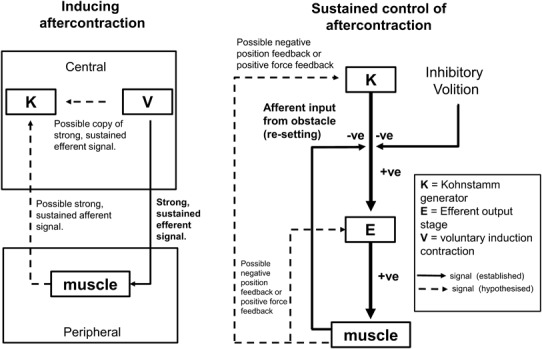
A model of the Kohnstamm phenomenon. The *left panel* shows a model of how an aftercontraction is induced from a strong, sustained voluntary contraction (*V*). Efferent output produces a contraction in the muscle, which will, upon relaxation (cessation of voluntary signal), display an aftercontraction. The Kohnstamm generator (*K*) is centrally located and must receive input during the induction. However, it is not known whether the necessary signal to the Kohnstamm generator originates from the muscle, and/or directly from central regions (*V*). The *right panel* shows how the aftercontraction is controlled once it has begun. The Kohnstamm generator (*K*) does not output directly to the muscle. Rather a positive signal is sent to an efferent output stage (*E* likely M1), which in turn produces the involuntary muscle contraction. The strength of the signal sent from the Kohnstamm generator can be reduced via both voluntary inhibition and via afferent signals resulting from the limb being arrested by a physical obstacle. While the limb is moving, it is not known if the Kohnstamm generator receives modulatory positive force feedback or negative position feedback from the muscle. Alternatively, this putative feedback might not modify the Kohnstamm generator directly, and instead operate at a lower level (*E*)

Figure 10. shows a model of the Kohnstamm phenomenon consistent with the reviewed literature. There is good evidence that the Kohnstamm generator is located centrally (De Havas et al. 2015; Duclos et al. 2007; Parkinson et al. 2009; Sapirstein et al. 1938). A strong, sustained isometric voluntary muscle contraction is necessary to induce the phenomenon (Fig. 10; left panel). This input shows a dose–response relationship, with greater input resulting in a lager output from the Kohnstamm generator (Allen 1937; Allen and O’Donoghue 1927; Brice and McDonagh 2001; Fessard and Tournay 1949). However, it is not known if the necessary input to the Kohnstamm generator arises centrally, from muscle afferents, or from other peripheral afferents. A hypothesised link between the Kohnstamm phenomenon and the TVR argues for afferent signals from muscle spindles being the necessary input (Duclos et al. 2007; Hagbarth and Nordin 1998). Further work is necessary to determine if this conjecture holds, and whether other afferent signals from the muscle (e.g., force signal from Golgi Tendon Organs) or from central regions, also contribute to establishing the Kohnstamm aftercontraction.

More is known about the sustained control of the aftercontraction (Fig. 10; right panel). Excitatory output from a central Kohnstamm generator can be reduced by afferent signals arising from the muscle in response to physical obstruction (Adamson and McDonagh 2004; De Havas et al. 2015). Importantly, this ‘gating’ of signal does not appear to modify the state of the Kohnstamm generator directly, as evidenced by the fact that a temporarily arrested arm, upon obstacle removal, rises to the same angle as an arm that was never arrested (De Havas et al. 2015). Similar findings have been observed for voluntary inhibition of the aftercontraction (De Havas et al. 2016; Fessard and Tournay 1949; Ghosh et al. 2014). It may be that other forms of afferent input (e.g. visual or vestibular input) can also modulate descending signals at this stage of the hierarchy (Bellincioni 1926; Wells 1944; Gilhodes et al. 1992). Excitatory output from the Kohnstamm generator appears to pass through an efferent output stage, which may be the locus of these modulations. This output stage may be located in M1, and may operate in the same manner as during voluntary movement (Duclos et al. 2007; Ghosh et al. 2014; Mathis et al. 1996). However, unlike during voluntary movements, during the Kohnstamm aftercontraction a copy of the efferent command is not compared against sensory inflow. This explains the subjectively strange feeling, which differs from the feeling of voluntary movements (Ghosh et al. 2014; De Havas et al. 2015, 2016). There is evidence that during an unobstructed aftercontraction the strength of the descending excitatory signal can be reduced by reducing the load on the muscle (Parkinson and McDonagh 2006). This reduction may reflect a positive force feedback loop between afferent signals from Golgi tendon organs and a central Kohnstamm generator. However, negative position feedback from muscle spindles could also explain the finding. It is not known if such afferent feedback loops modify the Kohnstamm generator directly or act at the efferent output level, nor whether they can have excitatory as well as inhibitory effects.

More work is needed to determine the control mechanisms underlying the Kohnstamm phenomenon. Nevertheless, the proposed model is an important step towards understanding the phenomenon and situating it within existing theories of motor control. On this account, the Kohnstamm phenomenon shares many features with voluntary control, including a hierarchical structure incorporating multiple levels of afferent feedback. The model highlights how voluntary commands and structures can be used to achieve movement without a feeling of ‘voluntariness’, calling into question traditional views on the distinctions between of voluntary and involuntary movement. By situating the Kohnstamm generator within a larger context of adaption and learning within the motor system, it may be possible to explore how the Kohnstamm phenomenon, like the lean aftereffect (Wright 2011), relates to normal postural control of the body.

## Outstanding questions

This review has identified the main currents of research in the Kohnstamm phenomenon over the previous century since it was first reported. Despite this body of knowledge, several key research questions remain. Here we briefly describe the questions that emerge, and could guide future research efforts.

Where is the Kohnstamm generator? Recent work has constrained theories, yet the exact anatomical location of the Kohnstamm generator remains unknown. It is also unclear if the Kohnstamm generator can be constrained to a single location or is better conceived of as multiple adaptions within the CNS.

What control principles govern the aftercontraction? Simple, purely central feedforward accounts seem unlikely, yet it is unclear what role negative-position feedback and positive-force feedback play in controlling the aftercontraction. Future work involving perturbations below the threshold for perception, and tasks where participants are instructed not to intervene in response to perturbations during the aftercontraction, could help answer this question.

Does afferent firing drive the aftercontraction? It remains unknown if muscle thixotropy and sustained afferent firing contribute to the aftercontraction or are merely incidental.

What signals during the induction establish the aftercontraction? The similarity of the Kohnstamm phenomenon to the TVR suggests muscle spindle signalling may establish central adaptions which produce the aftercontraction. However, this has not been tested and GTO or efferent signals may be crucial instead. How these signals are integrated by the putative Kohnstamm generator is also unknown.

Why does the Kohnstamm phenomenon feel so strange? The subjective feeling of the aftercontraction may be due to a lack of efference copies to cancel against the sensory inflow. However, it is not clear why the Kohnstamm feels so different to passive movements or whether the lack of efference copy account can truly explain all the subjective phenomena reported by participants.

What is the functional role of the Kohnstamm generator during “normal movement”? The Kohnstamm phenomenon may relate to postural control and share some features with rhythmic movements governed by central pattern generators. Yet there remains a large theoretical gap that needs to be bridged to convincingly link putative Kohnstamm generators to normal postural function.

## Conclusion

After many years of only intermittent study, the Kohnstamm phenomenon is now gradually gaining attention. Increasingly, the evidence is moving away from purely central or purely peripheral models. Instead, hybrid models are necessary, to account for both central adaptions and their interactions with sensory inflow. Modern electrophysiology and imaging have made the first steps towards elucidating the location of the putative Kohnstamm generator and towards comparing the aftercontraction to voluntary movement. There have also been attempts in recent years to quantify the subjective experience of the Kohnstamm phenomenon and relate the experience to the underlying control principles. However, despite the long history of study much remains unknown regarding the Kohnstamm phenomenon (see outstanding questions). Understanding the Kohnstamm phenomenon will inform the study of how body posture is maintained and provide novel insights to larger questions regarding motor control and the subjective awareness of movement.
